# The Link Between Dietary Indices, Sarcopenia, and Clinical Parameters in Diabetic and Non-Diabetic Hemodialysis Patients

**DOI:** 10.3390/jcm15134923

**Published:** 2026-06-24

**Authors:** Yahya Faruk Karatas, Gulsum Gizem Topal, Damla Gumus, Mevlude Kizil

**Affiliations:** 1 Department of Nutrition and Dietetics, Faculty of Health Sciences, Osmaniye Korkut Ata University, Osmaniye 80000, Turkey; 2 Department of Nutrition and Dietetics, Faculty of Health Sciences, Akdeniz University, Antalya 07070, Turkey; 3 Department of Nutrition and Dietetics, Faculty of Health Sciences, Hecettepe Univesity, Sıhhiye, Ankara 06100, Turkey

**Keywords:** dietary insulin index, dietary glycemic index, dietary glycemic load, dietary insulin load, sarcopenia, chronic kidney disease, diabetes mellitus

## Abstract

**Background and Objectives**: Sarcopenia is highly prevalent among maintenance hemodialysis (HD) patients, particularly in the presence of diabetes mellitus (DM). Dietary glycemic and insulinemic characteristics may contribute to metabolic disturbances associated with muscle deterioration, although evidence in HD populations remains limited. This study aimed to investigate the associations between dietary indices, sarcopenia, nutritional status, and clinical outcomes in diabetic (DM+) and non-diabetic (DM−) HD patients. **Materials and Methods**: This cross-sectional study included 92 maintenance HD patients (43 DM+ and 49 DM−). Dietary intake was assessed using three-day food records, and dietary insulin index (DII), dietary insulin load (DIL), dietary glycemic index (DGI), and dietary glycemic load (DGL) were calculated. Sarcopenia was evaluated using handgrip strength, bioelectrical impedance analysis, gait speed, and SARC-F. Anthropometric, biochemical, nutritional, and sarcopenia-related parameters were compared across tertiles of dietary indices. **Results**: Sarcopenia was identified in 32.6% of patients with diabetes and 36.7% of those without diabetes. Diabetic patients exhibited significantly lower handgrip strength, slower walking speed, longer walking time, and higher SARC-F scores (*p* < 0.01). Across DGL tertiles in DM+ patients, significant progressive increases were observed in body weight (*p* < 0.05), body mass index (*p* < 0.05), lean mass (*p* < 0.05), mid-upper arm circumference (*p* < 0.01), and triceps skinfold thickness (*p* < 0.01). Higher DIL and DGL tertiles were also associated with elevated serum phosphorus, LDL cholesterol, triglycerides, and total cholesterol levels (*p* < 0.05). DIL and DGL showed stronger associations with overall energy and nutrient intake compared with DII and DGI. However, no significant associations were identified between dietary indices and sarcopenia diagnosis or sarcopenia-related risk indicators after adjustment for age and sex. **Conclusions**: Dietary indices were associated with various anthropometric, biochemical, and nutritional parameters in HD patients, with more pronounced associations observed among patients with DM, suggesting a potential role of dietary quality in the nutritional and metabolic profile of this population.

## 1. Introduction

Chronic kidney disease (CKD), especially at the end-stage renal disease (ESRD) stage, remains a serious public health concern worldwide, carrying substantial morbidity and mortality risks. Metabolic disturbances, protein-energy wasting (PEW), systemic inflammation, and progressive muscle loss frequently co-occur in patients receiving hemodialysis (HD). Sarcopenia is a frequent clinical finding in this population, and the progressive decline in muscle mass and physical strength has been consistently associated with increased mortality risk [1,2].

The development of sarcopenia in HD patients is driven by multiple overlapping mechanisms, among which uremic toxin accumulation, metabolic acidosis, hormonal dysregulation, and insulin resistance appear to play particularly prominent roles. As a key anabolic hormone, insulin governs muscle protein turnover by promoting synthesis and limiting proteolysis. In the HD population, however, insulin resistance disrupts this balance, shifting muscle protein metabolism toward a net catabolic state and thereby accelerating muscle loss [3,4]. Diabetes mellitus (DM) constitutes one of the most prevalent comorbidities in the HD population and appears to compound the sarcopenic process through several distinct pathways. Chronic hyperglycemia, pre-existing insulin resistance, and diabetes-related vascular complications collectively impair muscle metabolism, and accumulating evidence suggests that the presence of DM substantially elevates sarcopenia risk in affected individuals [5,6].

Nutrition is one of the most important modifiable factors influencing sarcopenia and clinical outcomes in HD patients. While resistance exercise combined with targeted nutritional interventions has shown promise in improving muscle mass and physical function, results across studies have been inconsistent, reflecting considerable heterogeneity in patient populations, intervention protocols, and outcome measures. This variability points to the likelihood that the metabolic impact of dietary composition rather than caloric or protein quantity alone may be a more decisive determinant of muscle health in this population [7,8,9].

In recent decades, dietary indices capturing glycemic and insulinemic responses to food intake have attracted growing interest in clinical nutrition research. The dietary glycemic index (DGI) and dietary glycemic load (DGL) characterize the postprandial glucose rise triggered by specific foods or overall dietary patterns, while the dietary insulin index (DII) and dietary insulin load (DIL) offer a broader perspective by accounting for the insulin-stimulating properties of not only carbohydrates but also proteins and fats [10,11]. Dietary patterns marked by elevated glycemic and insulinemic responses have been linked to a cluster of metabolic disturbances, including hyperinsulinemia, peripheral insulin resistance, and enhanced lipogenesis, as shown in previous studies [12,13,14].

Dietary glycemic load has been identified as a significant predictor of oxidative stress and systemic inflammation in maintenance hemodialysis (MHD) patients, independent of adipocytokine levels and body composition. These results suggest that dietary nutrient quality plays an important role in the metabolic profile of MHD patients and may contribute to the modulation of chronic metabolic disturbances in this population [12]. In patients with type 2 diabetes, higher DII and DIL scores have been linked to increased cardiometabolic risk and a greater likelihood of developing metabolic syndrome. Given that dietary composition directly influences postprandial insulin responses, monitoring and managing insulinemic load may represent a practical approach to reducing the burden of secondary metabolic complications in this population [13].

Despite growing research on dietary indices and metabolic outcomes, evidence directly linking glycemic and insulinemic dietary indices to sarcopenia in HD patients remains scarce. Beyond dietary quantity, the metabolic impact of diet may be equally relevant in this context, and studies simultaneously examining both glycemic and insulinemic indices in relation to sarcopenia across diabetic and non-diabetic HD subgroups are still lacking. To address this gap, the present study aimed to investigate the associations between dietary indices (DII, DIL, DGI, and DGL), sarcopenia, and clinical parameters in both diabetic and non-diabetic HD patients.

## 2. Materials and Methods

### 2.1. Study Design and Setting

This study was designed as a cross-sectional analysis examining the relationships between dietary insulinemic and glycemic indices, sarcopenia, and clinical parameters in HD patients. Participants were recruited from the hemodialysis unit of Osmaniye Training and Research Hospital over a one-year period spanning September 2023 to September 2024. The study was conducted in accordance with the Declaration of Helsinki and approved by the Hacettepe University Health Sciences Research Ethics Committee (approval no: GO 23/492). Written informed consent was obtained from each participant before data collection.

### 2.2. Participants

The study population comprised patients with ESRD receiving maintenance HD. A total of 92 participants aged 18 years or older were enrolled in the study, out of whom 43 participants had a diagnosis of DM, while 49 did not. Eligible patients had been undergoing HD at least three times per week for a minimum of three months, with each session lasting 3.5–4 h and had a confirmed diagnosis of ESRD defined as a glomerular filtration rate (GFR) below 15 mL/min/1.73 m^2^. Participants were also required to ambulate independently and have Turkish literacy.

Exclusion criteria were established to ensure the validity of physical performance assessments and to minimize confounding. Bedridden patients, those with amputations or significant upper or lower extremity disabilities, and individuals unable to complete physical performance tests were excluded. Patients with malignancy, advanced liver disease, severe cardiovascular disease, chronic obstructive pulmonary disease, autoimmune or rheumatologic conditions, acute infections, HIV/AIDS, tuberculosis, neurological disorders including Alzheimer’s or Parkinson’s disease, and decompensated thyroid dysfunction were also ineligible. Patients with Type 1 DM, gestational DM, or secondary forms of DM were excluded, as were those with drug-induced muscle loss or long-term use of anti-inflammatory or hormonal agents known to affect muscle metabolism. Further exclusion criteria included conditions limiting mobility unrelated to diabetes, presence of implanted electronic devices, pregnancy, athlete status, and unwillingness to participate.

### 2.3. Data Collection

A structured questionnaire covering sociodemographic characteristics, medical history, and dietary habits was administered to participants through face-to-face interviews. Daily macro- and micronutrient intake was determined from three-day food records completed by each participant, encompassing the day preceding dialysis, the day of dialysis, and the day following dialysis.

#### 2.3.1. Dietary Assessment and Calculation of Dietary Indices

Food intake was recorded over three days: the day before dialysis, the day of dialysis, and the day after dialysis. Before data collection, participants were interviewed to ensure that they understood how to complete the food records. A Food and Nutrition Photo Catalog [15] was provided to assist with estimating portion sizes, along with a written guide on how to fill out the recording forms.

The Nutrition Information System (BEBIS 8.0) was used to analyze dietary intake data, and the DII, DIL, DGI, and DGL were subsequently derived from these analyses. These indices were used to evaluate postprandial insulin and glucose responses to dietary intake.

GI and II values were compiled from internationally validated reference sources. GI values were obtained from international tables of glycemic index and glycemic load values, using glucose as the reference standard (GI = 100) [16]. II values were determined according to the methodology of Holt et al., in which white bread providing 1000 kJ (approximately 239 kcal) serves as the reference food (II = 100) [10].

The DGI was calculated by multiplying the carbohydrate content (g) of each food by its GI value and dividing the total by daily carbohydrate intake. Glycemic load (GL) was computed for each food item using Equation (1) [16], and the total daily GL was obtained by summing the GL values of all foods consumed throughout the day. The DIL was estimated by weighting the II of each food against its energy content relative to the 239 kcal reference portion, as described in Equation (2) [10]. The DII was subsequently calculated by dividing the sum of energy weighted II values by total daily energy intake (Equation (3)) [17,18].

GL = Σ [(GI × Carbohydrate (g))/100](1)

IL = Σ [II × (Food Energy (kcal)/239 kcal)](2)

DII = [Σ (II × Food Energy)]/Σ Total Daily Energy(3)

Participants were then ranked into tertiles (T1–T3) based on each index, with the lowest tertile (T1) serving as the reference category.

#### 2.3.2. Assessment of Sarcopenia

Sarcopenia was assessed in accordance with the European Working Group on Sarcopenia in Older People (EWGSOP2) consensus [19]. All sarcopenia related assessments were performed by the same investigator, and the EWGSOP2 Find-Assess-Confirm-Severity (F-A-C-S) algorithm was applied retrospectively to the complete dataset in order to classify participants according to their sarcopenia status.

Initially sarcopenia risk was screened with the SARC-F questionnaire, which evaluates strength, walking ability, ability to rise from a chair, stair climbing, and history of falls, and a total score ≥ 4 was considered indicative of increased risk [20]. Muscle strength was measured by handgrip strength using a calibrated Takei hand dynamometer (Takei Scientific Instruments Co., Niigata, Japan). To avoid interference from vascular access, the measurement was obtained from the arm contralateral to the HD catheter or arteriovenous fistula. With the participant seated and the elbow flexed at 90°, three trials were performed and the highest value was recorded. Low muscle strength was defined as a handgrip value below 27 kg in men and below 16 kg in women.

Muscle mass was assessed by bioelectrical impedance analysis using a Tanita 545-N body composition device (Tanita Corporation, Tokyo, Japan). To limit the effect of fluid status on impedance values, measurements were obtained within 30 min after the mid-week HD session. Appendicular skeletal muscle mass (ASM) was defined as the sum of lean soft tissue mass of both upper and lower extremities obtained by bioelectrical impedance analysis, and the skeletal muscle mass index (SMI) was calculated as ASM divided by height squared in accordance with the EWGSOP2 recommendations. Low muscle mass was defined as SMI < 7.0 kg/m^2^ in men and <5.5 kg/m^2^ in women.

Physical performance was evaluated with the 4-m usual gait speed test, conducted according to the NIH protocol [21]. The mean of two trials was used, and a gait speed ≤ 0.8 m/s was considered indicative of low physical performance.

Sarcopenia-related quality of life was assessed with the SarQoL questionnaire, a validated self-administered instrument scored from 0 to 100, with higher scores indicating better quality of life [22].

After completion of all measurements, the EWGSOP2 algorithm was applied to the full dataset. SARC-F was used as a screening tool to identify individuals at risk for sarcopenia. Participants with low muscle strength were considered to have probable sarcopenia, and those with both low muscle strength and low SMI were classified as having confirmed sarcopenia.

#### 2.3.3. Assessment of Malnutrition

Malnutrition risk and nutritional status was evaluated using the seven-point subjective global assessment (SGA-7p) [23]. The SGA-7p comprises seven components: weight loss over the preceding six months, dietary intake, gastrointestinal symptoms persisting for more than two weeks, functional capacity, disease severity in relation to nutritional requirements, muscle wasting, and fat store depletion. Based on these components, a single global score was assigned to each participant. Scores of 6–7 indicate well-nourished status, scores of 3–5 correspond to mild-to-moderate malnutrition, and scores of 1–2 denote severe malnutrition [23].

#### 2.3.4. Anthropometric Measurements

All anthropometric measurements were carried out by the researcher in accordance with standardized procedures. Height was recorded using a stadiometer with participants barefoot and the head positioned in the Frankfort plane. Body mass index (BMI) was calculated by dividing body weight in kilograms by the square of height in meter [24]. Waist circumference was measured in the standing position using a rigid tape measure at the midpoint between the lowest rib and the iliac crest, with the tape held parallel to the floor. Body weight and composition were assessed with a TANITA 545N portable BIA device (Tanita Corporation, Tokyo, Japan). Mid-upper arm circumference (MUAC) and triceps skinfold thickness (TSF) were obtained using a non-elastic tape measure and a skinfold caliper, respectively. Handgrip strength was measured with a hand dynamometer.

#### 2.3.5. Biochemical Parameters

Biochemical parameters were retrieved from routine clinical follow-up records, and no additional blood samples were collected for this study. These included serum albumin, total protein, glucose, uric acid, calcium, phosphorus, sodium, C-reactive protein (CRP), ferritin, total iron-binding capacity (TIBC), high-density lipoprotein (HDL), low-density lipoprotein (LDL), triglycerides, and total cholesterol, all of which were measured prior to the midweek dialysis session as part of standard clinical practice. Blood urea nitrogen (BUN), serum creatinine, and potassium levels were recorded both before and after the dialysis session. Dialysis adequacy was evaluated using the Kt/V index.

### 2.4. Statistical Analysis

All statistical analyses were carried out using SPSS version 23.0 (IBM Corp., Armonk, NY, USA). Continuous variables are expressed as mean ± SD and categorical variables as *n* (%). Participants were stratified by diabetes status and classified into tertiles (T1–T3) according to DII, DIL, DGI, and DGL scores. Anthropometric and sarcopenia-related continuous variables were compared across tertiles using one-way ANCOVA with age and sex as covariates, with Bonferroni-corrected pairwise ANCOVA for post hoc comparisons. Biochemical parameters and dietary intake variables were also compared across tertiles using one-way ANCOVA adjusted for age and sex, with Bonferroni-corrected pairwise ANCOVA as post hoc tests. Categorical variables were examined using the chi-square test, with Bonferroni-corrected pairwise chi-square or Fisher’s exact tests for post hoc comparisons when the overall test was significant (*p* < 0.05).

The associations between dietary index tertiles and sarcopenia risk indicators, including sarcopenia diagnosis, SGA-7p category, and SARC-F score, were examined using binary logistic regression, with T1 serving as the reference category. Three binary outcomes were defined: sarcopenia diagnosis, above-median SGA-7p score, and above-median SARC-F score. Both unadjusted and adjusted odds ratios (ORs) with 95% confidence intervals (CIs) are presented. Model 2 was adjusted for age and sex, whereas Model 3 was additionally adjusted for BMI, HD duration, and CRP. In participants with diabetes, diabetes duration was included as an additional covariate. A linear trend across tertiles was tested by entering the tertile variable as a continuous term in the model. Statistical significance was defined as *p* < 0.05.

## 3. Results

### 3.1. Demographic, Clinical, and Anthropometric Characteristics of the Participants

The demographic, clinical, and anthropometric characteristics of the participants stratified by tertiles of DII and DIL across DM+ and DM− patients are presented in Table 1. Non-stratified findings for diabetic and non-diabetic HD patients are provided in the Appendix A. The mean age of the participants was 60.19 ± 13.03 and 51.65 ± 18.22 years in the DM+ and DM− groups, respectively. The age distribution was comparable across tertiles of both DII and DIL within each subgroup, with no statistically significant differences observed. Sex distribution varied significantly across DII tertiles in the DM+ group (*p* = 0.049), while dialysis-related parameters were largely similar across tertiles in both groups. Anthropometric measurements did not differ significantly across DII or DIL tertiles, except for MUAC, which showed a significant variation across DIL tertiles in the DM+ group (*p* = 0.040). Sarcopenia-related parameters revealed marked differences between DM+ and DM− patients. Handgrip strength was significantly lower in DM+ (*p* < 0.05), accompanied by longer walking time, lower walking speed, and higher SARC-F scores (*p* < 0.001) (Table A1). Despite these differences in functional performance, the prevalence of sarcopenia diagnosis, quality of life scores, and nutritional status indicators did not significantly differ across tertiles or between groups.

Participant characteristics stratified by tertiles of DGI and DGL are shown in Table 2. No significant differences were observed in age, sex distribution, or dialysis-related variables across DGI and DGL tertiles in either subgroup. Anthropometric characteristics demonstrated more pronounced variations across DGL tertiles in the DM+ group. Specifically, body weight (*p* = 0.039), MUAC (*p* = 0.002), triceps skinfold thickness (*p* = 0.007), hip circumference (*p* = 0.045), lean mass (*p* = 0.031), and BMI *(p* = 0.014) increased progressively across DGL tertiles, indicating a gradient in body composition associated with higher DGL. In contrast, no consistent differences were observed across DGI tertiles or within the non-diabetic group. Regarding sarcopenia-related parameters, no significant differences were detected across DGI or DGL tertiles within subgroups. Nevertheless, consistent differences between DM+ and DM− patients persisted. Handgrip strength remained significantly lower in diabetic individuals (*p* < 0.05), while walking time was longer, walking speed was lower, and SARC-F scores were higher compared to non-diabetic patients (Table A1). The prevalence of sarcopenia diagnosis, SarQoL scores, and SGA-7p did not differ significantly across DGI or DGL tertiles or between groups.

### 3.2. Biochemical Characteristics of the Participants Across Tertiles of Dietary Indices

The biochemical characteristics of the participants across tertiles of DII and DIL are presented in Table 3. In the DM+ group, several biochemical parameters exhibited significant variations across the DII and DIL tertiles. Notably, the serum phosphorus levels increased significantly across DII tertiles (*p* = 0.005), with higher levels observed in the upper tertiles. Similarly, phosphorus concentrations also differed across DIL tertiles (*p* = 0.008), indicating a consistent pattern associated with higher insulinemic dietary exposure. In addition, the total protein levels showed a borderline significant decrease across DII tertiles (*p* = 0.050). Significant differences were also observed in several biochemical markers across increasing DIL tertiles. Serum albumin levels were higher in the intermediate and upper tertiles compared to the lowest tertile (*p* = 0.032). Pre-dialysis creatinine levels increased across tertiles (*p* = 0.019), while uric acid (*p* = 0.043) and calcium levels (*p* = 0.017) also showed significant variations. Lipid parameters, including LDL cholesterol (*p* = 0.011) and total cholesterol (*p* = 0.027), increased across DIL tertiles, suggesting a relationship between higher DIL and lipid metabolism. In contrast, most biochemical parameters in the DM− group did not differ significantly across DII or DIL tertiles. However, as expected, fasting glucose levels were markedly higher in DM+ patients compared to DM− individuals, confirming the metabolic distinction between the groups (*p* < 0.001) (Table A2).

Table 4 details the biochemical profiles of participants across tertiles of DGI and DGL. Among DM+, DGL tertiles were associated with several significant biochemical differences. Serum albumin levels differed across tertiles (*p* = 0.044), while post-dialysis creatinine also varied significantly (*p* = 0.003). In addition, the phosphorus levels increased across DGL tertiles (*p* = 0.010), indicating a pattern consistent with that observed for DII and DIL indices. Lipid parameters demonstrated a clear gradient across DGL tertiles. LDL cholesterol (*p* = 0.012), triglycerides (*p* = 0.024), and total cholesterol (*p* = 0.005) increased significantly from the lowest to the highest tertile, suggesting a potential association between higher DGL and an adverse lipid profile in DM+. Conversely, DGI tertiles were not associated with significant differences in most biochemical parameters, indicating a weaker relationship compared to DGL (*p* > 0.05). Similarly, no consistent differences were observed across tertiles in the DM− group for either DGI or DGL (*p* > 0.05). Fasting glucose levels were significantly higher in the DM+ patients compared to DM− patients (*p* < 0.001), whereas other biochemical parameters did not differ significantly between groups after adjustment (Table A2).

### 3.3. Dietary Energy and Nutrient Intake

The distribution of dietary energy and nutrient intake according to DII and DIL tertiles is shown in Table 5. In the DM+ group, no significant differences were observed across DII tertiles for total energy or macronutrient intake (*p* > 0.05). In contrast, dietary intake patterns varied significantly across DIL tertiles. The total energy intake increased significantly across DIL tertiles (*p* = 0.004), accompanied by higher carbohydrate intake (*p* = 0.002) and protein intake (*p* < 0.001). In addition to macronutrients, several micronutrients demonstrated significant variations across DIL tertiles. Total fiber (*p* = 0.014), insoluble fiber (*p* = 0.020), and soluble fiber (borderline, *p* = 0.053) increased with higher DIL. Similarly, intake of B-group vitamins, including thiamine (*p* < 0.001) and riboflavin (*p* < 0.001), as well as folate (*p* = 0.031), showed significant increases. Mineral intake, including sodium (*p* = 0.002), potassium (*p* = 0.002), calcium (*p* < 0.001), magnesium (*p* = 0.005), phosphorus (*p* < 0.001), iron (*p* = 0.008), and zinc (*p* = 0.001), also increased across DIL tertiles. Among the DM− patients, similar trends were observed across DIL tertiles. Energy (*p* < 0.001), carbohydrate (*p* < 0.001), protein (*p* = 0.001), and protein intake per kilogram (*p* < 0.001) increased significantly. Fiber intake, including total (*p* = 0.014), soluble (*p* = 0.034), and insoluble fiber (*p* = 0.022), also demonstrated significant increases. In parallel, micronutrient intake, particularly B vitamins, calcium, phosphorus, potassium, and zinc, followed a similar upward trend across tertiles. In contrast, DII tertiles were not associated with significant differences in most dietary intake variables in either subgroup (*p* > 0.05).

Dietary energy and nutrient intake stratified by tertiles of DGI and DGL are summarized in Table 6. Consistent with findings for DIL, DGL tertiles were strongly associated with dietary intake patterns. In the DM+ group, energy intake increased significantly across DGL tertiles (*p* < 0.001), along with carbohydrate intake (*p* < 0.001) and protein intake (*p* < 0.001). Total fat intake also differed across tertiles (*p* = 0.033), while fiber intake, including total (*p* = 0.021) and soluble fiber (*p* = 0.003), showed significant increases. In addition, several micronutrients demonstrated significant variation across DGL tertiles. Intakes of potassium (*p* = 0.032), calcium (*p* = 0.023), magnesium (*p* = 0.015), phosphorus (*p* = 0.003), iron (*p* = 0.005), and zinc (*p* = 0.009) were significantly higher in the upper tertiles. Similar to DIL, these findings indicate that higher DGL is associated with increased overall nutrient intake. Among the DM− group, DGL tertiles were likewise associated with significant increases in energy intake (*p* = 0.007) and carbohydrate intake (*p* < 0.001), along with trends toward higher protein and micronutrient intake, although these were less consistent compared to the DM+ group. In contrast, DGI tertiles showed limited associations with dietary intake variables. Only a limited number of nutrients, namely insoluble fiber (*p* = 0.010), fructose (*p* = 0.005), vitamin B6 (*p* = 0.026), and vitamin C (*p* = 0.018), differed significantly across tertiles in DM+ patients, whereas no consistent pattern emerged in the DM− group. Moreover, no significant differences were observed in dietary energy and nutrient intakes between DM+ and DM− participants (*p* > 0.05) (Table A3).

### 3.4. Associations Between Dietary Indices and Sarcopenia-Related Outcomes

Associations between dietary indices and sarcopenia-related outcomes were further examined in both DM+ and DM− patients (Table 7 and Table 8, respectively). In DM+ patients, no significant associations were observed between tertiles of DII, DIL, DGI, or DGL and sarcopenia diagnosis, SGA-7p or SARC-F in either crude or adjusted models. Similarly, in DM− patients, dietary indices were not significantly associated with sarcopenia-related outcomes, although higher odds ratios were observed in the upper tertiles of some indices, particularly DIL and DGL, without significant trend patterns.

## 4. Discussion

The present study investigated the associations of dietary insulin and glycemic related indices with sarcopenia, nutritional status, and clinical parameters in diabetic and non-diabetic HD patients. Sarcopenia was diagnosed in 34.5% of the study population, including 32.6% of patients with diabetes and 36.7% of those without diabetes. The prevalence of sarcopenia falls within the range reported in recent international studies, including 25% in Brazil [25], 42.4% in Tunisia [26], 35.6% in Japan [27], and 54% in China [28]. In the present study, the DM+ patients exhibited more pronounced impairments in muscle function and physical performance compared with non-diabetic individuals. Lower handgrip strength, slower walking performance, and higher SARC-F scores observed among the DM+ group may reflect the combined associations of insulin resistance, chronic inflammation, oxidative stress, and diabetes-related metabolic dysregulation, all of which have been implicated in accelerated muscle deterioration in CKD populations [29,30,31,32]. These findings further support the thought that declines in muscle strength and functional capacity may occur earlier and more prominently than measurable reductions in muscle mass in HD patients [26]. In addition, the observed increases in body weight, BMI, lean mass, and anthropometric measurements across higher DGL tertiles in DM+ patients may indicate that DGL is associated with overall body composition characteristics. The lack of significant associations between DGI/DGL tertiles and sarcopenia prevalence also suggests that glycemic dietary exposure alone may be insufficient to explain the multifactorial mechanisms underlying sarcopenia development in the HD population.

Another notable finding of the present study was the stronger association of DIL and DGL, particularly among DM+ patients, with several biochemical parameters related to metabolic and nutritional status. Higher DIL and DGL tertiles were accompanied by increases in serum phosphorus, lipid parameters, creatinine, and albumin levels, suggesting that dietary load-based indices may reflect broader metabolic and dietary exposure in HD patients. The observed elevations in phosphorus levels across higher DIL and DGL tertiles may be clinically relevant, given the well-established relationship between hyperphosphatemia, cardiovascular risk, and mortality in patients undergoing HD [33,34]. Similarly, the less favorable lipid profile observed across higher DGL tertiles in DM+ patients may indicate an interaction between glycemic dietary burden and altered lipid metabolism in the context of DM and CKD [35]. In contrast, the relatively limited associations observed for DII and DGI may suggest that load-based indices more effectively capture the cumulative metabolic impact of dietary intake than index-based measures alone.

The dietary intake findings of the present study further demonstrated that DIL and DGL were more strongly associated with overall energy and nutrient intake than DII and DGI. Higher DIL and DGL tertiles were consistently accompanied by greater energy, carbohydrate, protein, fiber, vitamin, and mineral intake in both DM+ and DM− patients, indicating that these indices may more comprehensively reflect the total dietary exposure and nutrient density. This distinction may be attributable to the fact that dietary load indices incorporate both the quality and quantity of carbohydrate-related dietary intake, thereby providing a broader representation of cumulative glycemic and insulinemic burden [36]. Notably, among the DM+ patients, mean protein intake in the lowest DIL tertile fell below the minimum intake of 1.2 g/kg body weight/day recommended by the KDOQI guidelines for metabolically stable HD patients [37]. Given the central role of protein-energy wasting in the pathogenesis of sarcopenia [38], this deficit may represent a clinically important nutritional vulnerability.

In contrast, the relatively limited associations observed for DII and DGI suggest that index-based measures alone may insufficiently capture the complexity of dietary intake patterns in HD patients. The parallel increases observed in micronutrients and minerals across higher DIL and DGL tertiles may additionally reflect greater overall food consumption rather than isolated changes in dietary composition. Nevertheless, the accompanying increases in phosphorus and sodium intake observed in higher tertiles may have important clinical implications in the HD population, in whom dietary management requires careful balancing between adequate nutritional intake and the restriction of nutrients associated with adverse clinical outcomes [39,40].

Despite the observed associations between dietary indices and dietary and biochemical parameters, no significant relationships were identified between DII, DIL, DGI, or DGL and sarcopenia diagnosis, nutritional status, or functional sarcopenia risk in either DM+ or DM− patients after adjustment for age, sex, BMI, dialysis vintage, and CRP. These findings may reflect the multifactorial nature of sarcopenia in patients undergoing HD and suggest that dietary indices alone may not adequately capture the complex mechanisms underlying sarcopenia in this population [41,42]. Another possible explanation for the lack of significant associations is the relatively small sample size, which may have limited the statistical power to detect modest associations. This issue is particularly relevant in dialysis-based research, as the prevalence of sarcopenia in maintenance HD populations, although substantial in absolute terms (ranging from approximately 24% to over 50% across recent reports), may still result in a limited number of events when participants are stratified into exposure tertiles within moderately sized samples [43,44,45].

This study has several strengths and limitations that should be considered when interpreting the findings. To the best of our current knowledge, this is among the limited studies simultaneously evaluating dietary insulin and glycemic related indices in relation to sarcopenia-related outcomes in both DM+ and DM− HD patients. Another important strength is the comprehensive assessment of anthropometric, biochemical, dietary, nutritional, and sarcopenia-related functional parameters based on EWGSOP2 criteria. In addition, the simultaneous evaluation of DII, DIL, DGI, and DGL allowed for a comparison between load-based and index-based dietary measures in a metabolically vulnerable population. However, the cross-sectional design precludes causal inference and the single-center design may limit the generalizability of the findings. Dietary intake was assessed using self-reported food records, which may be subject to recall bias and reporting inaccuracies. Another limitation of the present study is the relatively small sample size of some subgroups resulting from stratification according to DM status and dietary index tertiles. Although these subgroup analyses provided clinically relevant insights, the limited number of participants in certain categories may have reduced the stability and precision of the statistical estimates. A further limitation is that body composition was assessed using BIA. Although measurements were performed after dialysis sessions to minimize the effects of fluid overload, residual fluid shifts in HD patients may have affected the BIA-derived estimates of muscle mass. In addition, advanced imaging techniques such as DXA, CT, and MRI were not available. Furthermore, information regarding the underlying etiology of kidney disease, previous corticosteroid therapy, and residual renal function was not available for all participants. Since these factors may influence nutritional status, body composition, and sarcopenia-related parameters, their potential confounding effects cannot be excluded. Future studies incorporating advanced imaging techniques and more comprehensive clinical data may provide a more comprehensive characterization of sarcopenia in patients undergoing HD.

## 5. Conclusions

The present study demonstrated that dietary glycemic and insulinemic related indices were associated with distinct nutritional, anthropometric, and biochemical patterns in HD patients, particularly among individuals with DM. DM+ patients exhibited poorer muscle function and physical performance, while DIL and DGL showed more pronounced associations with dietary intake characteristics and metabolic parameters than index-based measures. These findings highlight the potential relevance of dietary glycemic and insulinemic exposure in the metabolic and nutritional profile of HD patients and suggest that load-based indices may provide a more comprehensive reflection of dietary burden in this population. Given the multifactorial nature of sarcopenia in HD patients, further prospective and longitudinal studies are warranted to better clarify the role of dietary glycemic and insulinemic characteristics in sarcopenia-related parameters and overall health outcomes.

## Figures and Tables

**Table 1:** Demographic and clinical characteristics of participants across tertiles of DII and DIL.

	DM+								DM−							
	DII				DIL				DII				DIL			
	T1 *n* = 13	T2 *n* = 15	T3 *n* = 15	*p*-Value	T1 *n* = 13	T2 *n* = 12	T3 *n* = 18	*p*-Value	T1 *n* = 18	T2 *n* = 15	T3 *n* = 16	*p*-Value	T1 *n* = 18	T2 *n* = 18	T3 *n* = 13	*p*-Value
Age (years)	57.38 ± 17.79	61.00 ± 11.32	61.80 ± 9.94	0.652	60.62 ± 16.89	62.42 ± 11.19	58.39 ± 11.39	0.712	56.17 ± 18.20	48.27 ± 18.10	49.75 ± 18.48	0.416	49.22 ± 18.36	58.44 ± 16.26	45.62 ± 18.93	0.119
Gender																
Male, n (%)	**3 (23.1%) ^a^**	**9 (60.0%) ^ab^**	**10 (66.7%) ^b^**	**0.049**	5 (38.5%)	4 (33.3%)	13 (72.2%)	0.062	6 (33.3%)	9 (60.0%)	8 (50.0%)	0.297	6 (33.3%)	8 (44.4%)	9 (69.2%)	0.137
Female, n (%)	**10 (76.9%) ^a^**	**6 (40.0%) ^b^**	**5 (33.3%) ^b^**	**0.049**	8 (61.5%)	8 (66.7%)	5 (27.8%)	0.062	12 (66.7%)	6 (40.0%)	8 (50.0%)	0.297	12 (66.7%)	10 (55.6%)	4 (30.8%)	0.137
Dialysis duration (years)	6.15 ± 3.98	4.53 ± 3.64	8.07 ± 6.01	0.132	4.77 ± 3.83	7.58 ± 4.68	6.44 ± 5.46	0.345	4.17 ± 3.40	8.40 ± 7.33	5.88 ± 3.12	0.055	5.94 ± 6.67	5.50 ± 2.81	6.85 ± 5.29	0.773
Anthropometric measurements																
Weight (kg)	68.53 ± 16.00	72.19 ± 15.86	72.89 ± 13.29	0.847	65.10 ± 13.51	75.04 ± 14.81	73.35 ± 15.09	0.219	70.00 ± 14.20	65.81 ± 13.87	66.53 ± 14.72	0.904	67.94 ± 14.65	72.07 ± 12.02	60.88 ± 14.41	0.248
MUAC (cm)	25.27 ± 6.77	26.93 ± 3.60	28.37 ± 4.80	0.299	**23.81 ± 4.88 ^a^**	**27.83 ± 5.11 ^b^**	**28.58 ± 4.62 ^b^**	**0.040**	25.88 ± 5.42	25.90 ± 3.48	26.05 ± 4.72	0.771	25.66 ± 5.16	27.56 ± 4.49	24.10 ± 3.09	0.361
TSF (mm)	17.54 ± 8.69	16.87 ± 6.03	18.10 ± 8.95	0.733	15.00 ± 6.02	16.25 ± 8.16	20.14 ± 8.25	0.059	15.00 ± 5.54	15.13 ± 7.90	17.50 ± 6.58	0.216	14.47 ± 5.15	18.78 ± 8.40	13.73 ± 4.22	0.141
Waist circumference (cm)	88.62 ± 17.28	90.50 ± 14.25	91.23 ± 14.56	0.942	82.65 ± 13.86	96.58 ± 16.82	91.36 ± 12.77	0.067	91.14 ± 12.35	85.40 ± 11.34	88.38 ± 13.00	0.647	87.69 ± 13.24	93.17 ± 10.68	83.08 ± 11.15	0.235
Hip circumference (cm)	90.96 ± 16.99	92.23 ± 14.32	91.10 ± 13.94	0.862	85.15 ± 14.98	98.29 ± 16.32	91.44 ± 11.81	0.087	94.50 ± 12.34	88.07 ± 9.91	87.94 ± 11.20	0.451	91.28 ± 12.22	94.94 ± 10.07	82.85 ± 8.84	0.081
Waist/hip ratio	0.97 ± 0.04	0.98 ± 0.05	1.00 ± 0.04	0.362	0.97 ± 0.05	0.98 ± 0.05	1.00 ± 0.04	0.644	0.96 ± 0.04	0.97 ± 0.08	1.00 ± 0.04	0.052	0.96 ± 0.05	0.98 ± 0.06	1.00 ± 0.05	0.390
Body fat (%)	29.51 ± 7.21	28.10 ± 8.78	28.64 ± 8.49	0.498	27.23 ± 7.39	32.45 ± 6.70	27.29 ± 8.89	0.219	29.65 ± 8.78	24.56 ± 10.35	25.36 ± 10.81	0.926	28.86 ± 10.85	28.96 ± 8.82	20.54 ± 8.31	0.380
Lean mass (kg)	20.62 ± 4.49	22.62 ± 4.00	23.56 ± 3.01	0.605	20.48 ± 3.80	22.44 ± 3.87	23.62 ± 3.77	0.201	21.23 ± 3.48	22.01 ± 3.48	20.84 ± 3.55	0.686	21.08 ± 3.31	22.09 ± 3.04	20.65 ± 4.22	0.162
BMI (kg/m^2^)	25.51 ± 5.37	25.99 ± 5.39	26.71 ± 5.60	0.631	24.08 ± 4.57	27.91 ± 5.71	26.34 ± 5.42	0.190	26.23 ± 5.04	24.09 ± 5.18	24.60 ± 5.15	0.888	25.48 ± 5.40	26.45 ± 4.67	22.48 ± 4.62	0.396
Sarcopenia-related measurements																
Hand grip strength (kg)	16.73 ± 4.14	18.73 ± 3.90	18.33 ± 4.47	0.643	16.31 ± 5.01	17.96 ± 3.86	19.22 ± 3.42	0.136	19.51 ± 5.53	20.85 ± 3.95	20.43 ± 4.70	0.799	20.00 ± 3.35	19.97 ± 6.24	20.87 ± 4.34	0.310
Walking time (s)	7.19 ± 1.46	8.84 ± 2.82	7.85 ± 3.03	0.081	8.16 ± 2.52	7.99 ± 2.58	7.88 ± 2.81	0.506	6.21 ± 1.06	5.82 ± 1.72	5.68 ± 1.74	0.801	5.94 ± 1.32	6.07 ± 1.61	5.67 ± 1.68	0.429
Walking speed (m/s)	0.58 ± 0.13	0.49 ± 0.14	0.60 ± 0.29	0.132	0.53 ± 0.15	0.57 ± 0.26	0.57 ± 0.21	0.415	0.66 ± 0.12	0.75 ± 0.23	0.77 ± 0.23	0.623	0.71 ± 0.19	0.71 ± 0.21	0.76 ± 0.21	0.386
SARC-F score	5.00 ± 2.12	5.60 ± 3.09	5.33 ± 2.99	0.631	5.62 ± 2.57	5.58 ± 2.50	4.94 ± 3.10	0.834	3.06 ± 1.39	2.80 ± 2.57	2.56 ± 2.48	0.806	3.00 ± 1.88	3.00 ± 2.50	2.31 ± 2.02	0.828
SQoL score	45.73 ± 7.97	43.44 ± 5.93	43.69 ± 5.43	0.396	43.12 ± 8.24	46.06 ± 4.87	43.60 ± 5.89	0.445	46.59 ± 7.05	51.29 ± 11.94	43.86 ± 0.0	0.915	50.09 ± 12.66	50.20 ± 5.50	43.96 ± 12.35	0.735
SGA-7p score	6.23 ± 1.24	5.87 ± 1.36	6.20 ± 0.56	0.576	6.00 ± 1.22	6.25 ± 0.62	6.06 ± 1.26	0.626	6.11 ± 0.83	6.27 ± 1.03	6.38 ± 0.89	0.859	6.17 ± 0.86	6.11 ± 1.02	6.54 ± 0.78	0.704
SGA-7p category																
Well-nourished	10 (76.9%)	10 (66.7%)	14 (93.3%)	0.359	9 (69.2%)	11 (91.7%)	14 (77.8%)	0.471	15 (83.3%)	11 (73.3%)	14 (87.5%)	0.580	15 (83.3%)	14 (77.8%)	11 (84.6%)	0.865
Mild to moderate malnutrition	3 (23.1%)	4 (26.7%)	1 (6.7%)	0.359	4 (30.8%)	1 (8.3%)	3 (16.7%)	0.471	3 (16.7%)	4 (26.7%)	2 (12.5%)	0.580	3 (16.7%)	4 (22.2%)	2 (15.4%)	0.865
Severe malnutrition	0 (0.0%)	1 (6.7%)	0 (0.0%)	0.359	0 (0.0%)	0 (0.0%)	1 (5.6%)	0.471	0 (0.0%)	0 (0.0%)	0 (0.0%)	0.580	0 (0.0%)	0 (0.0%)	0 (0.0%)	0.865
Sarcopenia diagnosis																
Present, n (%)	3 (23.1%)	5 (33.3%)	6 (40.0%)	0.633	3 (23.1%)	4 (33.3%)	7 (38.9%)	0.649	5 (27.8%)	7 (46.7%)	6 (37.5%)	0.532	5 (27.8%)	7 (38.9%)	6 (46.2%)	0.562
Absent, n (%)	10 (76.9%)	10 (66.7%)	9 (60.0%)	0.633	10 (76.9%)	8 (66.7%)	11 (61.1%)	0.649	13 (72.2%)	8 (53.3%)	10 (62.5%)	0.532	13 (72.2%)	11 (61.1%)	7 (53.8%)	0.562

Data are presented as mean ± SD for continuous variables and *n* (%) for categorical variables. Age was compared across tertiles using one-way ANOVA with Tukey HSD post hoc test; dialysis duration was compared using one-way ANOVA with Bonferroni-corrected independent *t*-tests. Anthropometric and sarcopenia-related continuous variables were compared using one-way ANCOVA adjusted for age and sex, with Bonferroni-corrected pairwise ANCOVA for post hoc comparisons. Categorical variables were analyzed using the chi-square test with Bonferroni-corrected pairwise comparisons. Bold values indicate statistically significant results (*p* < 0.05). Different superscript letters (a, b indicate statistically significant differences between groups, whereas groups sharing at least one letter are not significantly different.

**Table 2:** Demographic and clinical characteristics of participants across tertiles of DGI and DGL.

	DM+								DM−							
	DGI				DGL				DGI				DGL			
	T1 *n* = 14	T2 *n* = 12	T3 *n* = 17	*p*-Value	T1 *n* = 12	T2 *n* = 14	T3 *n* = 17	*p*-Value	T1 *n* = 17	T2 *n* = 18	T3 *n* = 14	*p*-Value	T1 *n* = 19	T2 *n* = 16	T3 *n* = 14	*p*-Value
Age (years)	59.29 ± 15.77	61.67 ± 13.39	59.88 ± 10.84	0.896	60.50 ± 15.76	61.50 ± 13.38	58.88 ± 11.22	0.858	49.41 ± 19.42	55.67 ± 20.43	49.21 ± 13.45	0.511	49.89 ± 20.53	55.00 ± 14.29	50.21 ± 19.66	0.678
Gender																
Male, n (%)	4 (28.6%)	6 (50.0%)	12 (70.6%)	0.066	3 (25.0%)	7 (50.0%)	12 (70.6%)	0.053	5 (29.4%)	9 (50.0%)	9 (64.3%)	0.145	8 (42.1%)	5 (31.2%)	10 (71.4%)	0.077
Female, n (%)	10 (71.4%)	6 (50.0%)	5 (29.4%)	0.066	9 (75.0%)	7 (50.0%)	5 (29.4%)	0.053	12 (70.6%)	9 (50.0%)	5 (35.7%)	0.145	11 (57.9%)	11 (68.8%)	4 (28.6%)	0.077
Dialysis duration (years)	4.71 ± 3.77	5.50 ± 4.15	8.06 ± 5.62	0.127	6.17 ± 4.06	6.29 ± 4.81	6.29 ± 5.54	0.997	5.82 ± 6.81	6.33 ± 4.91	5.86 ± 2.57	0.949	4.95 ± 4.34	6.69 ± 5.95	6.71 ± 5.08	0.511
Anthropometric measurements																
Weight (kg)	66.44 ± 16.10	74.05 ± 16.84	73.44 ± 11.81	0.433	**63.93 ± 10.17 ^a^**	**68.98 ± 14.20 ^b^**	**78.49 ± 15.52 ^c^**	**0.039**	68.46 ± 15.55	64.42 ± 16.13	70.60 ± 8.46	0.227	65.73 ± 12.44	72.46 ± 16.48	64.54 ± 12.73	0.359
MUAC (cm)	25.93 ± 4.24	27.12 ± 7.13	27.62 ± 4.37	0.764	**23.00 ± 4.15 ^a^**	**27.04 ± 4.61 ^ab^**	**29.62 ± 4.66 ^b^**	**0.002**	26.09 ± 5.71	25.93 ± 4.94	25.77 ± 2.24	0.924	25.37 ± 4.58	27.11 ± 4.86	25.38 ± 4.30	0.696
TSF (mm)	16.93 ± 6.07	18.08 ± 9.90	17.56 ± 7.84	0.751	**13.75 ± 3.76 ^a^**	**16.64 ± 7.73 ^ab^**	**20.85 ± 8.83 ^b^**	**0.007**	16.82 ± 7.09	15.19 ± 7.34	15.54 ± 5.29	0.735	13.82 ± 5.20	17.69 ± 7.42	16.54 ± 7.11	0.175
Waist circumference (cm)	87.75 ± 15.89	92.58 ± 18.56	90.50 ± 11.78	0.762	83.00 ± 11.08	89.04 ± 14.94	96.21 ± 15.69	0.060	89.24 ± 13.48	86.14 ± 13.97	90.57 ± 7.86	0.289	85.97 ± 9.98	92.06 ± 15.52	87.79 ± 10.61	0.478
Hip circumference (cm)	90.71 ± 14.66	94.12 ± 19.96	90.18 ± 10.52	0.754	**85.67 ± 10.93 ^a^**	**90.32 ± 16.05 ^ab^**	**96.47 ± 14.92 ^b^**	**0.045**	92.94 ± 12.81	87.28 ± 12.23	91.29 ± 8.16	0.161	89.63 ± 8.86	94.50 ± 15.52	86.71 ± 8.01	0.449
Waist/hip ratio	0.97 ± 0.05	0.99 ± 0.05	1.00 ± 0.04	0.683	0.97 ± 0.04	0.99 ± 0.05	1.00 ± 0.04	0.954	0.96 ± 0.05	0.99 ± 0.06	0.99 ± 0.05	0.649	0.96 ± 0.06	0.97 ± 0.05	1.01 ± 0.05	0.104
Body fat (%)	29.25 ± 7.74	29.71 ± 9.97	27.57 ± 7.14	0.628	**27.42 ± 6.38 ^a^**	**28.96 ± 8.70 ^b^**	**29.43 ± 8.89 ^c^**	**0.044**	27.86 ± 12.11	25.54 ± 10.77	26.74 ± 5.93	0.261	26.28 ± 10.46	30.60 ± 9.66	22.77 ± 8.72	0.636
Lean mass (kg)	20.04 ± 3.60	22.94 ± 4.40	23.81 ± 3.14	0.153	**19.68 ± 3.27 ^a^**	**21.79 ± 3.51 ^ab^**	**24.67 ± 3.49 ^b^**	**0.031**	20.79 ± 3.85	20.72 ± 3.25	22.81 ± 3.00	0.240	21.11 ± 3.35	21.63 ± 3.68	21.32 ± 3.59	0.351
BMI (kg/m^2^)	25.23 ± 5.80	26.72 ± 6.41	26.37 ± 4.30	0.580	**23.67 ± 3.15 ^a^**	**25.48 ± 5.46 ^ab^**	**28.32 ± 5.84 ^b^**	**0.014**	25.82 ± 5.47	23.76 ± 6.05	25.74 ± 2.76	0.175	24.43 ± 4.43	27.01 ± 6.01	23.62 ± 4.41	0.411
Sarcopenia-related measurements																
Hand grip strength (kg)	17.46 ± 3.88	17.67 ± 4.74	18.65 ± 4.14	0.805	16.75 ± 4.09	16.82 ± 4.51	19.82 ± 3.40	0.269	18.53 ± 4.86	19.88 ± 3.86	22.71 ± 4.93	0.239	20.25 ± 4.01	19.04 ± 5.86	21.52 ± 4.25	0.804
Walking time (s)	8.77 ± 3.08	7.32 ± 1.87	7.84 ± 2.60	0.149	7.74 ± 1.76	8.43 ± 3.14	7.82 ± 2.72	0.467	5.84 ± 1.52	6.25 ± 1.35	5.58 ± 1.69	0.818	6.00 ± 1.41	5.90 ± 1.67	5.83 ± 1.55	0.468
Walking speed (m/s)	0.51 ± 0.18	0.60 ± 0.24	0.57 ± 0.20	0.362	0.54 ± 0.13	0.55 ± 0.26	0.57 ± 0.20	0.840	0.74 ± 0.22	0.67 ± 0.15	0.78 ± 0.23	0.617	0.71 ± 0.20	0.73 ± 0.22	0.73 ± 0.19	0.408
SARC-F score	5.64 ± 3.05	5.33 ± 2.50	5.06 ± 2.77	0.941	5.42 ± 2.35	6.07 ± 2.46	4.65 ± 3.16	0.494	3.00 ± 2.18	3.11 ± 2.05	2.21 ± 2.22	0.739	2.89 ± 1.94	3.06 ± 2.54	2.43 ± 1.99	0.930
SQoL score	43.06 ± 5.39	42.62 ± 6.43	47.36 ± 6.88	0.636	45.17 ± 7.02	43.97 ± 6.31	43.15 ± 5.87	0.445	54.79 ± 8.80	45.14 ± 9.14	43.86 ± 0.0	0.746	50.85 ± 10.45	48.73 ± 6.89	43.96 ± 12.35	0.727
SGA-7p score	5.71 ± 1.33	6.17 ± 1.19	6.35 ± 0.70	0.072	6.17 ± 1.27	5.71 ± 1.27	6.35 ± 0.70	0.267	6.12 ± 0.93	6.28 ± 0.75	6.36 ± 1.08	0.841	6.11 ± 0.88	6.19 ± 1.05	6.50 ± 0.76	0.595
SGA-7p category																
Well-nourished	9 (64.3%)	10 (83.3%)	15 (88.2%)	0.424	9 (75.0%)	10 (71.4%)	15 (88.2%)	0.536	13 (76.5%)	15 (83.3%)	12 (85.7%)	0.782	15 (78.9%)	13 (81.2%)	12 (85.7%)	0.883
Mild to moderate malnutrition	4 (28.6%)	2 (16.7%)	2 (11.8%)	0.424	3 (25.0%)	3 (21.4%)	2 (11.8%)	0.536	4 (23.5%)	3 (16.7%)	2 (14.3%)	0.782	4 (21.1%)	3 (18.8%)	2 (14.3%)	0.883
Severe malnutrition	1 (7.1%)	0 (0.0%)	0 (0.0%)	0.424	0 (0.0%)	1 (7.1%)	0 (0.0%)	0.536	0 (0.0%)	0 (0.0%)	0 (0.0%)	0.782	0 (0.0%)	0 (0.0%)	0 (0.0%)	0.883
Sarcopenia diagnosis																
Present, n (%)	4 (28.6%)	5 (41.7%)	5 (29.4%)	0.729	3 (25.0%)	6 (42.9%)	5 (29.4%)	0.587	4 (23.5%)	10 (55.6%)	4 (28.6%)	0.110	7 (36.8%)	4 (25.0%)	7 (50.0%)	0.366
Absent, n (%)	10 (71.4%)	7 (58.3%)	12 (70.6%)	0.729	9 (75.0%)	8 (57.1%)	12 (70.6%)	0.587	13 (76.5%)	8 (44.4%)	10 (71.4%)	0.110	12 (63.2%)	12 (75.0%)	7 (50.0%)	0.366

Data are presented as mean ± SD for continuous variables and *n* (%) for categorical variables. Age was compared across tertiles using one-way ANOVA with the Tukey HSD post hoc test; dialysis duration was compared using one-way ANOVA with Bonferroni-corrected independent *t*-tests. Anthropometric and sarcopenia-related continuous variables were compared using one-way ANCOVA adjusted for age and sex, with Bonferroni-corrected pairwise ANCOVA for post hoc comparisons. Categorical variables were analyzed using the chi-square test with Bonferroni-corrected pairwise comparisons. Bold values indicate statistically significant results (*p* < 0.05). Different superscript letters (a, b, c) indicate statistically significant differences between groups, whereas groups sharing at least one letter are not significantly different.

**Table 3:** Biochemical parameters of the participants across tertiles of DII and DIL.

	DM+								DM−							
	DII				DIL				DII				DIL			
	T1 *n* = 13	T2 *n* = 15	T3 *n* = 15	*p*-Value	T1 *n* = 13	T2 *n* = 12	T3 *n* = 18	*p*-Value	T1 *n* = 18	T2 *n* = 15	T3 *n* = 16	*p*-Value	T1 *n* = 18	T2 *n* = 18	T3 *n* = 13	*p*-Value
Albumin (g/dL)	38.02 ± 4.12	39.62 ± 2.48	39.02 ± 1.94	0.169	**37.31 ± 3.78 ^a^**	**39.98 ± 2.12 ^b^**	**39.39 ± 2.27 ^b^**	**0.032**	40.40 ± 2.56	41.24 ± 3.79	39.78 ± 2.12	0.343	41.38 ± 2.80	39.69 ± 3.05	40.23 ± 2.55	0.376
Total protein (g/dL)	**70.06 ± 9.02 ^a^**	**65.48 ± 3.91 ^b^**	**64.21 ± 7.65 ^b^**	**0.050**	68.05 ± 9.64	67.36 ± 4.32	64.63 ± 7.04	0.432	72.14 ± 21.66	65.83 ± 5.69	65.88 ± 4.38	0.594	67.52 ± 5.33	71.55 ± 21.83	64.38 ± 3.90	0.656
Fasting glucose (mg/dL)	189.08 ± 57.26	236.59 ± 97.43	209.33 ± 42.34	0.111	220.83 ± 78.46	183.67 ± 59.01	226.22 ± 71.49	0.142	108.39 ± 20.78	117.43 ± 28.09	115.14 ± 29.18	0.607	111.63 ± 27.79	121.14 ± 27.47	104.99 ± 18.01	0.193
BUN before (mg/dL)	130.30 ± 27.77	139.71 ± 32.05	142.92 ± 23.02	0.885	134.92 ± 27.84	126.93 ± 27.73	147.57 ± 25.70	0.440	135.59 ± 32.50	137.81 ± 27.60	143.34 ± 15.01	0.632	137.53 ± 24.53	134.02 ± 32.27	147.16 ± 16.48	0.918
BUN after (mg/dL)	29.63 ± 12.39	41.51 ± 14.05	40.61 ± 11.48	0.268	32.80 ± 11.77	34.84 ± 15.62	42.92 ± 11.87	0.505	37.08 ± 13.98	37.51 ± 14.23	37.39 ± 11.34	0.715	34.07 ± 12.83	38.55 ± 13.40	40.09 ± 12.64	0.682
Creatinine before (mg/dL)	7.41 ± 1.48	8.06 ± 1.28	8.07 ± 1.17	0.635	**7.02 ± 1.04 ^a^**	**7.74 ± 0.88 ^ab^**	**8.56 ± 1.39 ^b^**	**0.019**	7.77 ± 1.60	8.16 ± 1.17	8.64 ± 1.89	0.385	7.81 ± 1.48	8.37 ± 1.69	8.40 ± 1.67	0.694
Creatinine after (mg/dL)	2.38 ± 0.68	2.97 ± 0.64	2.71 ± 0.34	0.116	2.39 ± 0.66	2.80 ± 0.53	2.86 ± 0.55	0.119	2.67 ± 0.69	2.65 ± 0.64	3.08 ± 0.71	0.117	2.59 ± 0.79	2.88 ± 0.59	2.98 ± 0.67	0.496
Uric acid (mg/dL)	5.69 ± 1.11	6.23 ± 0.78	5.79 ± 0.51	0.243	**5.54 ± 0.51 ^a^**	**6.26 ± 1.29 ^b^**	**5.96 ± 0.52 ^a^**	**0.043**	6.20 ± 0.86	6.10 ± 0.81	5.99 ± 0.67	0.591	6.15 ± 0.84	6.10 ± 0.80	6.04 ± 0.69	0.686
Calcium (mg/dL)	8.77 ± 0.32	8.68 ± 0.54	8.76 ± 0.32	0.811	**8.50 ± 0.35 ^a^**	**8.74 ± 0.41 ^ab^**	**8.90 ± 0.36 ^b^**	**0.017**	8.62 ± 0.41	8.76 ± 0.55	8.52 ± 0.57	0.407	8.71 ± 0.49	8.70 ± 0.44	8.42 ± 0.60	0.171
Phosphorus (mg/dL)	**4.15 ± 1.10 ^a^**	**5.05 ± 1.21 ^b^**	**5.01 ± 0.83 ^b^**	**0.005**	**4.17 ± 1.17 ^a^**	**4.58 ± 0.64 ^ab^**	**5.32 ± 1.09 ^b^**	**0.008**	5.01 ± 1.12	4.61 ± 1.27	5.25 ± 0.89	0.229	5.04 ± 1.19	4.57 ± 1.05	5.42 ± 0.95	0.190
Sodium (mEq/L)	137.69 ± 2.18	137.30 ± 2.66	138.80 ± 1.70	0.219	137.88 ± 2.72	137.67 ± 1.83	138.17 ± 2.26	0.961	137.44 ± 2.06	137.13 ± 2.20	137.00 ± 2.31	0.811	137.11 ± 2.05	136.72 ± 2.19	138.00 ± 2.16	0.221
Potassium before (mEq/L)	5.19 ± 0.76	5.79 ± 0.82	5.30 ± 0.63	0.126	5.40 ± 0.94	5.14 ± 0.73	5.65 ± 0.62	0.239	5.18 ± 0.70	5.45 ± 0.78	5.53 ± 0.75	0.646	5.25 ± 0.88	5.35 ± 0.51	5.58 ± 0.82	0.968
Potassium after (mEq/L)	3.57 ± 0.26	3.79 ± 0.31	3.70 ± 0.31	0.286	3.64 ± 0.22	3.67 ± 0.31	3.75 ± 0.35	0.640	3.56 ± 0.24	3.57 ± 0.36	3.63 ± 0.23	0.711	3.54 ± 0.37	3.59 ± 0.18	3.65 ± 0.23	0.829
CRP (mg/L)	11.47 ± 14.56	10.28 ± 10.23	7.56 ± 5.53	0.733	14.24 ± 14.99	7.26 ± 4.68	8.03 ± 8.48	0.160	11.14 ± 10.58	13.26 ± 20.09	7.22 ± 5.01	0.424	10.11 ± 11.50	11.99 ± 18.05	9.01 ± 5.50	0.862
Ferritin (ng/mL)	704.23 ± 432.88	588.07 ± 196.84	601.47 ± 153.23	0.289	697.69 ± 439.01	553.67 ± 176.22	626.89 ± 161.98	0.442	675.06 ± 242.38	556.33 ± 220.15	582.69 ± 196.38	0.470	647.06 ± 195.87	638.78 ± 263.53	513.38 ± 181.03	0.414
TIBC (µg/dL)	173.12 ± 90.36	150.69 ± 46.67	148.01 ± 24.07	0.162	168.52 ± 91.62	150.85 ± 38.86	151.67 ± 35.76	0.634	152.85 ± 45.40	152.15 ± 58.54	173.82 ± 74.56	0.396	143.97 ± 41.89	151.98 ± 47.82	191.35 ± 83.69	0.282
HDL (mg/dL)	39.47 ± 5.55	35.40 ± 9.27	44.19 ± 16.27	0.141	39.42 ± 8.43	39.06 ± 9.90	40.32 ± 15.17	0.994	42.53 ± 16.73	35.03 ± 7.88	40.00 ± 15.03	0.519	35.87 ± 12.10	44.26 ± 17.62	37.58 ± 9.23	0.281
LDL (mg/dL)	80.25 ± 17.27	99.49 ± 26.06	91.41 ± 18.92	0.083	**76.40 ± 17.30 ^a^**	**91.11 ± 15.61 ^b^**	**101.13 ± 24.00 ^b^**	**0.011**	86.83 ± 23.00	79.23 ± 20.24	87.08 ± 31.55	0.752	86.34 ± 21.72	89.17 ± 32.34	75.81 ± 15.91	0.493
Triglycerides (mg/dL)	105.24 ± 48.05	169.65 ± 91.61	144.74 ± 78.59	0.065	107.90 ± 47.84	178.39 ± 100.08	141.14 ± 73.63	0.091	149.20 ± 91.41	139.59 ± 56.44	115.36 ± 63.72	0.381	169.93 ± 85.07	116.76 ± 56.73	112.69 ± 61.58	0.061
Total cholesterol (mg/dL)	144.30 ± 23.41	167.99 ± 34.77	165.98 ± 29.05	0.087	**140.38 ± 22.85 ^a^**	**168.08 ± 26.74 ^b^**	**169.08 ± 33.09 ^b^**	**0.027**	160.08 ± 24.88	144.77 ± 26.89	150.68 ± 33.73	0.665	158.44 ± 26.15	158.90 ± 34.55	134.73 ± 13.84	0.151
Kt/V	1.75 ± 0.23	1.53 ± 0.34	1.53 ± 0.22	0.550	1.70 ± 0.20	1.62 ± 0.44	1.51 ± 0.16	0.494	1.60 ± 0.26	1.65 ± 0.42	1.64 ± 0.48	0.879	1.75 ± 0.38	1.50 ± 0.21	1.65 ± 0.53	0.237

Data are presented as mean ± SD. Biochemical parameters were compared across tertiles using one-way ANCOVA adjusted for age and sex. When the overall *p*-value was significant (*p* < 0.05), post hoc pairwise comparisons were conducted using Bonferroni-corrected pairwise ANCOVA. Bold values indicate statistically significant results (*p* < 0.05). Different superscript letters (a, b) indicate statistically significant differences between groups, whereas groups sharing at least one letter are not significantly different.

**Table 4:** Biochemical parameters of the participants across tertiles of DIL and DGL.

	DM+								DM−							
	DGI			DGL					DGI				DGL			
	T1 *n* = 14	T3 *n* = 12	T3 *n* = 17	*p*-Value	T1 *n* = 12	T3 *n* = 14	T3 *n* = 17	*p*-Value	T1 *n* = 17	T3 *n* = 18	T3 *n* = 14	*p*-Value	T1 *n* = 19	T3 *n* = 16	T3 *n* = 14	*p*-Value
Albumin (g/dL)	39.70 ± 2.97	37.82 ± 3.87	39.07 ± 1.89	0.301	**37.51 ± 3.79 ^a^**	**40.13 ± 2.88 ^b^**	**38.94 ± 1.77 ^a^**	**0.044**	40.44 ± 3.04	40.83 ± 2.87	39.99 ± 2.81	0.477	**41.88 ± 2.83 ^a^**	**39.41 ± 3.13 ^b^**	**39.72 ± 1.79 ^b^**	**0.025**
Total protein (g/dL)	65.75 ± 5.20	69.14 ± 9.00	65.06 ± 7.49	0.358	69.63 ± 9.10	67.29 ± 5.20	63.45 ± 6.71	0.064	72.23 ± 22.45	65.21 ± 4.63	67.04 ± 4.61	0.330	72.88 ± 21.04	65.34 ± 3.54	65.00 ± 4.94	0.129
Fasting glucose (mg/dL)	209.93 ± 100.85	216.88 ± 57.56	212.07 ± 52.64	0.903	208.90 ± 61.97	185.14 ± 73.68	238.12 ± 70.14	0.066	113.52 ± 31.57	111.49 ± 17.94	115.58 ± 28.22	0.851	112.86 ± 27.35	113.47 ± 21.23	113.92 ± 29.96	0.977
BUN before (mg/dL)	121.92 ± 22.32	142.89 ± 31.26	147.75 ± 24.34	0.100	133.15 ± 21.60	133.19 ± 30.04	145.35 ± 29.41	0.737	136.48 ± 29.82	135.59 ± 25.10	145.74 ± 22.62	0.845	138.78 ± 26.06	130.97 ± 31.78	147.76 ± 15.34	0.663
BUN after (mg/dL)	31.19 ± 13.66	37.89 ± 14.10	42.68 ± 11.22	0.430	28.78 ± 9.09	39.41 ± 15.09	42.35 ± 12.28	0.198	35.46 ± 13.69	37.98 ± 11.64	38.70 ± 14.39	0.945	35.38 ± 13.51	36.13 ± 13.63	41.29 ± 11.44	0.810
Creatinine before (mg/dL)	7.45 ± 1.19	7.80 ± 1.64	8.25 ± 1.10	0.634	6.95 ± 0.99	8.20 ± 1.02	8.23 ± 1.46	0.059	7.91 ± 1.21	7.99 ± 1.56	8.72 ± 2.00	0.577	7.86 ± 1.63	8.11 ± 1.02	8.67 ± 2.04	0.648
Creatinine after (mg/dL)	2.63 ± 0.74	2.66 ± 0.70	2.79 ± 0.41	0.907	**2.28 ± 0.59 ^a^**	**3.07 ± 0.54 ^b^**	**2.70 ± 0.48 ^a^**	**0.003**	2.63 ± 0.58	2.73 ± 0.62	3.10 ± 0.85	0.362	2.65 ± 0.77	2.67 ± 0.51	3.16 ± 0.69	0.261
Uric acid (mg/dL)	5.81 ± 0.94	6.09 ± 1.07	5.88 ± 0.56	0.489	5.52 ± 0.44	6.24 ± 1.20	5.93 ± 0.57	0.148	6.09 ± 0.63	5.99 ± 0.97	6.26 ± 0.69	0.659	6.08 ± 0.82	6.18 ± 0.85	6.04 ± 0.68	0.574
Calcium (mg/dL)	8.75 ± 0.45	8.66 ± 0.47	8.77 ± 0.32	0.760	8.75 ± 0.32	8.58 ± 0.48	8.85 ± 0.36	0.179	8.78 ± 0.45	8.38 ± 0.54	8.77 ± 0.43	0.050	8.70 ± 0.49	8.69 ± 0.45	8.46 ± 0.59	0.453
Phosphorus (mg/dL)	4.46 ± 0.65	4.56 ± 1.66	5.17 ± 0.84	0.104	**4.24 ± 1.26 ^a^**	**4.52 ± 0.64 ^ab^**	**5.34 ± 1.10 ^b^**	**0.010**	4.99 ± 0.95	4.72 ± 1.26	5.26 ± 1.08	0.470	4.65 ± 1.02	5.09 ± 1.31	5.26 ± 0.92	0.173
Sodium (mEq/L)	137.29 ± 2.92	137.46 ± 1.83	138.82 ± 1.67	0.223	138.00 ± 1.95	137.75 ± 2.64	138.06 ± 2.25	0.824	136.88 ± 1.50	137.67 ± 2.06	137.00 ± 2.88	0.605	137.00 ± 2.29	136.88 ± 1.89	137.86 ± 2.25	0.482
Potassium before (mEq/L)	5.24 ± 0.83	5.60 ± 0.82	5.48 ± 0.69	0.625	5.10 ± 0.67	5.71 ± 0.93	5.45 ± 0.63	0.197	5.44 ± 0.84	5.28 ± 0.72	5.42 ± 0.67	0.294	5.28 ± 0.85	5.31 ± 0.54	5.58 ± 0.78	0.821
Potassium after (mEq/L)	3.65 ± 0.39	3.75 ± 0.22	3.69 ± 0.27	0.793	3.55 ± 0.22	3.78 ± 0.32	3.73 ± 0.32	0.178	3.57 ± 0.36	3.57 ± 0.22	3.63 ± 0.24	0.911	3.52 ± 0.33	3.64 ± 0.24	3.63 ± 0.22	0.327
CRP (mg/L)	9.30 ± 8.71	12.67 ± 15.96	7.91 ± 6.12	0.562	13.84 ± 14.72	7.84 ± 7.30	8.29 ± 8.61	0.459	11.96 ± 19.14	7.83 ± 5.41	12.19 ± 11.31	0.553	8.75 ± 9.43	14.37 ± 19.79	8.49 ± 5.52	0.441
Ferritin (ng/mL)	584.00 ± 223.84	685.50 ± 440.18	623.29 ± 154.11	0.723	750.17 ± 419.30	522.71 ± 201.41	628.12 ± 164.74	0.079	713.82 ± 222.60	578.83 ± 164.09	518.93 ± 251.09	0.110	610.84 ± 240.73	663.50 ± 223.02	542.64 ± 193.85	0.671
TIBC (µg/dL)	142.34 ± 44.59	186.57 ± 85.34	147.02 ± 35.89	0.124	165.75 ± 94.16	151.59 ± 40.33	154.10 ± 36.76	0.702	151.15 ± 67.25	142.27 ± 44.91	191.73 ± 57.90	0.099	142.56 ± 43.82	156.90 ± 49.95	185.40 ± 80.56	0.307
HDL (mg/dL)	37.67 ± 8.35	37.32 ± 5.99	43.04 ± 16.28	0.387	39.69 ± 5.52	41.42 ± 10.49	38.28 ± 15.83	0.699	45.14 ± 16.49	36.08 ± 11.71	36.73 ± 12.23	0.101	38.66 ± 14.66	40.47 ± 15.06	39.20 ± 12.97	0.949
LDL (mg/dL)	94.75 ± 22.99	84.54 ± 21.29	92.11 ± 22.52	0.466	**79.51 ± 17.55 ^a^**	**85.67 ± 16.02 ^ab^**	**103.14 ± 24.33 ^b^**	**0.012**	78.06 ± 25.17	85.13 ± 20.65	91.80 ± 29.65	0.134	82.55 ± 20.40	94.94 ± 31.33	75.51 ± 19.83	0.194
Triglycerides (mg/dL)	157.67 ± 104.88	129.58 ± 56.08	136.56 ± 69.57	0.664	**98.36 ± 41.89 ^a^**	**142.29 ± 75.19 ^ab^**	**171.28 ± 90.35 ^b^**	**0.024**	123.67 ± 48.21	131.07 ± 88.39	154.54 ± 78.38	0.368	138.29 ± 63.22	152.46 ± 91.21	111.31 ± 60.77	0.353
Total cholesterol (mg/dL)	164.99 ± 31.43	151.09 ± 28.47	162.50 ± 32.45	0.486	**138.35 ± 18.46 ^a^**	**159.49 ± 22.27 ^ab^**	**176.02 ± 35.20 ^b^**	**0.005**	151.30 ± 26.15	145.73 ± 28.94	162.04 ± 30.88	0.075	150.95 ± 26.30	166.56 ± 27.93	137.90 ± 26.81	0.095
Kt/V	1.67 ± 0.41	1.61 ± 0.18	1.52 ± 0.20	0.967	1.77 ± 0.19	1.58 ± 0.37	1.49 ± 0.19	0.268	1.71 ± 0.36	1.56 ± 0.28	1.61 ± 0.51	0.767	1.71 ± 0.30	1.58 ± 0.34	1.58 ± 0.52	0.576

Data are presented as mean ± SD. Biochemical parameters were compared across tertiles using one-way ANCOVA adjusted for age and sex. When the overall *p*-value was significant (*p* < 0.05), post hoc pairwise comparisons were conducted using Bonferroni-corrected pairwise ANCOVA. Bold values indicate statistically significant results (*p* < 0.05). Different superscript letters (a, b) indicate statistically significant differences between groups, whereas groups sharing at least one letter are not significantly different.

**Table 5:** Dietary energy and nutrient intake of participants across tertiles of DII and DIL.

	DM+								DM−							
	DII				DIL				DII				DIL			
	T1 *n* = 13	T3 *n* = 15	T3 *n* = 15	*p*-Value	T1 *n* = 13	T3 *n* = 12	T3 *n* = 18	*p*-Value	T1 *n* = 18	T3 *n* = 15	T3 *n* = 16	*p*-Value	T1 *n* = 18	T3 *n* = 18	T3 *n* = 13	*p*-Value
Energy (kcal)	1623.61 ± 295.07	1683.68 ± 248.58	1703.53 ± 287.89	0.965	**1474.06 ± 240.46 ^a^**	**1654.23 ± 238.31 ^ab^**	**1827.87 ± 223.42 ^b^**	**0.004**	1637.54 ± 311.56	1664.54 ± 249.77	1741.27 ± 328.81	0.643	**1535.71 ± 217.80 ^a^**	**1588.88 ± 234.23 ^a^**	**2004.75 ± 224.91 ^b^**	**<0.001**
Carbohydrate (g)	184.19 ± 38.67	205.98 ± 36.10	219.99 ± 45.01	0.211	**172.55 ± 27.09 ^a^**	**200.12 ± 39.91 ^ab^**	**229.96 ± 36.00 ^b^**	**0.002**	188.89 ± 45.27	198.27 ± 42.08	224.01 ± 39.81	0.091	**183.81 ± 38.96 ^a^**	**188.59 ± 29.02 ^a^**	**250.38 ± 35.02 ^b^**	**<0.001**
Protein (g)	61.29 ± 18.26	61.87 ± 12.50	64.60 ± 13.40	0.880	**49.45 ± 8.48 ^a^**	**63.41 ± 8.40 ^b^**	**71.67 ± 14.19 ^b^**	**<0.001**	58.21 ± 16.94	61.90 ± 10.97	63.89 ± 15.01	0.681	**53.76 ± 13.11 ^a^**	**59.49 ± 11.57 ^a^**	**73.85 ± 12.59 ^b^**	**0.001**
Protein (g/kg)	0.91 ± 0.19	0.89 ± 0.21	0.92 ± 0.28	0.912	0.80 ± 0.22	0.87 ± 0.15	1.01 ± 0.24	0.053	0.86 ± 0.27	0.98 ± 0.26	1.01 ± 0.33	0.575	**0.82 ± 0.23 ^a^**	**0.85 ± 0.22 ^a^**	**1.25 ± 0.24 ^b^**	**<0.001**
Total fat (g)	69.55 ± 12.70	65.89 ± 12.69	60.78 ± 10.89	0.129	63.51 ± 16.26	64.77 ± 12.51	66.73 ± 9.14	0.830	70.23 ± 15.34	67.47 ± 12.08	63.38 ± 17.69	0.336	63.24 ± 9.27	64.51 ± 17.96	76.20 ± 15.07	0.064
PUFA (g)	13.31 ± 4.78	13.00 ± 4.76	11.29 ± 3.40	0.274	13.69 ± 5.70	10.80 ± 3.14	12.77 ± 3.71	0.293	14.91 ± 7.26	13.06 ± 4.01	11.45 ± 4.68	0.119	12.82 ± 2.21	13.51 ± 8.41	13.35 ± 4.68	0.920
MUFA (g)	26.05 ± 6.14	24.34 ± 5.37	22.55 ± 4.58	0.196	24.31 ± 6.36	24.33 ± 6.82	24.11 ± 3.75	0.975	28.12 ± 6.18	24.78 ± 4.96	23.47 ± 8.95	0.174	25.37 ± 6.32	23.57 ± 6.77	28.66 ± 7.85	0.068
Saturated fat (g)	25.23 ± 5.38	23.95 ± 5.24	22.64 ± 4.87	0.493	21.27 ± 4.80	24.89 ± 4.66	25.08 ± 5.25	0.062	21.92 ± 5.97	24.82 ± 4.29	23.90 ± 5.47	0.563	**20.64 ± 4.12 ^a^**	**22.44 ± 4.59 ^a^**	**28.76 ± 4.20 ^b^**	**<0.001**
Cholesterol (mg)	206.54 ± 90.67	240.57 ± 112.16	262.68 ± 164.53	0.740	187.88 ± 99.81	216.52 ± 93.75	288.51 ± 148.62	0.284	257.24 ± 103.27	268.43 ± 169.78	254.66 ± 86.65	0.973	229.58 ± 85.68	274.45 ± 159.58	281.46 ± 97.91	0.587
Total fiber (g)	20.27 ± 5.47	22.22 ± 6.12	19.77 ± 4.18	0.442	**17.22 ± 3.67 ^a^**	**21.79 ± 6.11 ^ab^**	**22.67 ± 4.62 ^b^**	**0.014**	21.30 ± 5.81	18.86 ± 5.88	20.28 ± 4.64	0.304	**20.48 ± 6.41 ^ab^**	**17.55 ± 3.71 ^a^**	**23.54 ± 4.33 ^b^**	**0.014**
Soluble fiber (g)	6.39 ± 1.92	7.24 ± 2.55	6.88 ± 1.48	0.699	5.77 ± 1.48	6.80 ± 2.02	7.68 ± 2.06	0.053	6.81 ± 2.44	6.15 ± 1.85	7.08 ± 1.44	0.312	**6.72 ± 2.54 ^ab^**	**5.86 ± 1.30 ^a^**	**7.83 ± 1.27 ^b^**	**0.034**
Insoluble fiber (g)	13.65 ± 3.68	14.72 ± 4.26	12.75 ± 3.01	0.353	**11.33 ± 2.57 ^a^**	**14.77 ± 4.36 ^ab^**	**14.73 ± 3.25 ^b^**	**0.020**	13.81 ± 3.81	12.34 ± 4.11	13.20 ± 3.30	0.349	**13.34 ± 4.12 ^ab^**	**11.39 ± 2.69 ^a^**	**15.37 ± 3.31 ^b^**	**0.022**
Fructose	13.75 ± 5.53	13.56 ± 6.11	8.57 ± 5.10	0.057	10.71 ± 5.12	12.83 ± 6.46	12.08 ± 6.43	0.562	11.71 ± 4.70	13.82 ± 5.39	13.68 ± 6.16	0.643	12.55 ± 4.27	11.16 ± 5.91	16.17 ± 5.01	0.073
Sucrose	13.08 ± 5.82	17.42 ± 6.30	15.34 ± 4.68	0.179	14.46 ± 7.06	14.95 ± 6.65	16.34 ± 4.11	0.880	16.08 ± 9.11	18.91 ± 13.43	17.90 ± 6.03	0.754	16.01 ± 8.34	16.40 ± 6.62	21.25 ± 14.07	0.217
Vitamin B1 (mg)	0.78 ± 0.17	0.79 ± 0.18	0.77 ± 0.16	0.835	**0.64 ± 0.10 ^a^**	**0.86 ± 0.14 ^b^**	**0.84 ± 0.16 ^b^**	**<0.001**	0.78 ± 0.20	0.69 ± 0.19	0.72 ± 0.18	0.233	**0.71 ± 0.18 ^ab^**	**0.67 ± 0.19 ^a^**	**0.85 ± 0.16 ^b^**	**0.024**
Vitamin B2 (mg)	1.25 ± 0.33	1.24 ± 0.25	1.20 ± 0.30	0.807	**0.98 ± 0.23 ^a^**	**1.31 ± 0.18 ^b^**	**1.36 ± 0.27 ^b^**	**<0.001**	1.17 ± 0.33	1.25 ± 0.18	1.22 ± 0.28	0.785	**1.08 ± 0.23 ^a^**	**1.19 ± 0.26 ^a^**	**1.42 ± 0.22 ^b^**	**0.002**
Vitamin B6 (mg)	1.18 ± 0.29	1.19 ± 0.27	0.98 ± 0.31	0.116	0.95 ± 0.29	1.19 ± 0.28	1.17 ± 0.29	0.067	1.17 ± 0.28	1.10 ± 0.33	1.02 ± 0.34	0.258	**1.07 ± 0.28 ^ab^**	**0.97 ± 0.32 ^a^**	**1.32 ± 0.26 ^b^**	**0.013**
Folate (µg)	312.55 ± 81.21	307.27 ± 82.14	262.54 ± 74.73	0.090	**242.68 ± 57.38 ^a^**	**300.24 ± 71.08 ^b^**	**325.15 ± 86.25 ^c^**	**0.031**	308.81 ± 57.38	272.51 ± 59.18	274.99 ± 80.29	0.263	**282.81 ± 60.63 ^ab^**	**262.85 ± 56.07 ^a^**	**324.94 ± 76.51 ^b^**	**0.006**
Vitamin C (mg)	107.81 ± 27.13	97.80 ± 47.41	78.12 ± 43.33	0.143	75.30 ± 33.07	98.25 ± 34.93	104.58 ± 48.35	0.151	**110.06 ± 35.26 ^a^**	**85.14 ± 44.80 ^ab^**	**74.22 ± 35.36 ^b^**	**0.025**	**110.38 ± 44.24 ^a^**	**67.23 ± 33.28 ^b^**	**96.05 ± 29.11 ^ab^**	**0.003**
Sodium (mg)	3832.76 ± 1069.77	4064.33 ± 759.15	4337.53 ± 1359.57	0.691	**3315.50 ± 728.44 ^a^**	**3904.48 ± 1113.78 ^ab^**	**4772.14 ± 870.04 ^b^**	**0.002**	3765.03 ± 884.90	3969.45 ± 812.99	3877.54 ± 855.24	0.960	**3416.07 ± 716.69 ^a^**	**3840.18 ± 821.66 ^ab^**	**4518.50 ± 611.21 ^b^**	**0.003**
Potassium (mg)	2225.02 ± 492.67	2228.36 ± 530.93	2094.17 ± 533.47	0.706	**1777.05 ± 307.46 ^a^**	**2337.98 ± 449.66 ^b^**	**2366.99 ± 521.16 ^b^**	**0.002**	2278.04 ± 416.53	2003.09 ± 460.86	2065.84 ± 544.44	0.182	**2090.13 ± 387.69 ^ab^**	**1925.32 ± 460.77 ^a^**	**2448.19 ± 483.73 ^b^**	**0.006**
Calcium (mg)	776.76 ± 237.32	781.46 ± 158.34	767.31 ± 179.59	0.956	**608.93 ± 154.56 ^a^**	**834.14 ± 143.16 ^b^**	**855.76 ± 162.69 ^b^**	**<0.001**	714.30 ± 235.48	801.46 ± 105.57	801.08 ± 190.64	0.381	**678.21 ± 171.82 ^a^**	**755.13 ± 171.43 ^a^**	**915.12 ± 157.87 ^b^**	**0.003**
Magnesium (mg)	237.71 ± 61.40	261.01 ± 58.65	247.08 ± 58.83	0.668	**205.71 ± 43.58 ^a^**	**268.63 ± 62.71 ^b^**	**267.44 ± 51.20 ^b^**	**0.005**	267.33 ± 69.87	237.15 ± 70.16	235.41 ± 57.57	0.218	247.94 ± 64.86	223.19 ± 69.29	281.18 ± 53.33	0.066
Phosphorus (mg)	1049.86 ± 275.47	1079.34 ± 219.17	1062.91 ± 209.92	0.975	**851.07 ± 138.53 ^a^**	**1117.70 ± 194.35 ^b^**	**1183.65 ± 199.81 ^b^**	**<0.001**	1015.30 ± 282.39	1048.65 ± 182.30	1053.35 ± 223.10	0.968	**935.21 ± 216.17 ^a^**	**997.97 ± 212.11 ^a^**	**1235.48 ± 157.34 ^b^**	**0.002**
Iron (mg)	8.20 ± 2.12	8.77 ± 2.05	8.57 ± 1.85	0.869	**7.10 ± 1.35 ^a^**	**9.08 ± 2.43 ^ab^**	**9.19 ± 1.50 ^b^**	**0.008**	9.12 ± 2.90	7.63 ± 2.17	8.08 ± 1.87	0.138	8.30 ± 2.80	7.64 ± 2.35	9.31 ± 1.64	0.185
Zinc (mg)	8.72 ± 2.40	8.66 ± 2.03	8.82 ± 2.09	0.979	**7.11 ± 1.60 ^a^**	**8.86 ± 1.53 ^b^**	**9.83 ± 2.12 ^b^**	**0.001**	7.88 ± 2.10	8.35 ± 1.45	8.52 ± 2.16	0.817	**7.23 ± 1.51 ^a^**	**8.01 ± 1.84 ^a^**	**9.91 ± 1.47 ^b^**	**0.001**

Data are presented as mean ± SD. Dietary intake variables were compared across tertiles using one-way ANCOVA adjusted for age and sex. When the overall *p*-value was significant (*p* < 0.05), post hoc pairwise comparisons were conducted using Bonferroni-corrected pairwise ANCOVA. Bold values indicate statistically significant results (*p* < 0.05). Different superscript letters (a, b) indicate statistically significant differences between groups, whereas groups sharing at least one letter are not significantly different.

**Table 6:** Dietary energy and nutrient intake of participants across tertiles of DGI and DGL.

	DM+								DM−							
	DGI				DGL				DGI				DGL			
	T1 *n* = 14	T3 *n* = 12	T3 *n* = 17	*p*-Value	T1 *n* = 12	T3 *n* = 14	T3 *n* = 17	*p*-Value	T1 *n* = 17	T3 *n* = 18	T3 *n* = 14	*p*-Value	T1 *n* = 19	T3 *n* = 16	T3 *n* = 14	*p*-Value
Energy (kcal)	1633.88 ± 236.05	1652.25 ± 295.87	1718.46 ± 292.47	0.967	**1576.99 ± 224.63 ^a^**	**1504.58 ± 240.54 ^a^**	**1878.07 ± 191.65 ^b^**	**<0.001**	1576.50 ± 202.19	1728.79 ± 312.20	1741.82 ± 356.47	0.374	**1555.89 ± 213.15 ^a^**	**1629.58 ± 306.80 ^ab^**	**1904.94 ± 273.16 ^b^**	**0.007**
Carbohydrate (g)	197.00 ± 36.94	196.39 ± 32.30	215.84 ± 50.35	0.711	**174.80 ± 29.97 ^a^**	**184.66 ± 26.48 ^a^**	**241.24 ± 31.12 ^b^**	**<0.001**	181.71 ± 34.04	212.92 ± 41.97	216.91 ± 50.94	0.057	**179.24 ± 39.71 ^a^**	**197.27 ± 28.49 ^a^**	**242.60 ± 39.84 ^b^**	**<0.001**
Protein (g)	57.18 ± 7.15	65.87 ± 20.38	64.88 ± 13.54	0.306	**56.16 ± 10.50 ^a^**	**55.45 ± 10.01 ^a^**	**73.15 ± 14.09 ^b^**	**<0.001**	58.98 ± 12.57	60.26 ± 14.75	65.09 ± 16.85	0.784	57.47 ± 12.92	58.28 ± 16.16	69.58 ± 12.14	0.158
Protein (g/kg)	0.90 ± 0.20	0.91 ± 0.27	0.91 ± 0.23	0.954	0.90 ± 0.19	0.83 ± 0.20	0.97 ± 0.27	0.374	0.89 ± 0.21	1.01 ± 0.37	0.93 ± 0.27	0.355	0.90 ± 0.23	0.86 ± 0.34	1.12 ± 0.26	0.114
Total fat (g)	66.46 ± 14.97	65.28 ± 12.53	64.13 ± 10.35	0.819	**70.93 ± 12.41 ^a^**	**58.73 ± 12.73 ^b^**	**66.51 ± 9.95 ^c^**	**0.033**	66.39 ± 12.68	68.53 ± 16.56	66.28 ± 17.20	0.865	65.89 ± 9.46	65.68 ± 20.41	70.53 ± 15.33	0.826
PUFA (g)	12.70 ± 4.85	11.33 ± 4.27	13.14 ± 4.01	0.603	**14.87 ± 4.59 ^a^**	**10.04 ± 3.69 ^b^**	**12.84 ± 3.75 ^ab^**	**0.007**	12.95 ± 3.77	13.10 ± 3.48	13.69 ± 9.26	0.992	12.95 ± 3.30	13.71 ± 8.48	13.01 ± 4.58	0.729
MUFA (g)	25.11 ± 6.53	23.58 ± 6.32	23.97 ± 3.76	0.735	25.98 ± 5.38	22.97 ± 6.89	24.05 ± 3.87	0.346	26.18 ± 7.57	25.87 ± 7.85	24.48 ± 5.58	0.828	26.08 ± 6.03	24.31 ± 7.10	26.36 ± 8.52	0.591
Saturated fat (g)	24.23 ± 4.94	25.39 ± 5.24	22.52 ± 5.19	0.397	24.93 ± 4.87	21.75 ± 4.89	24.88 ± 5.28	0.170	22.58 ± 4.99	24.55 ± 6.52	23.10 ± 4.20	0.504	22.27 ± 4.42	22.69 ± 7.09	25.93 ± 3.49	0.323
Cholesterol (mg)	187.52 ± 74.24	253.45 ± 123.74	268.65 ± 154.36	0.382	198.64 ± 93.31	219.29 ± 95.42	281.17 ± 159.39	0.570	252.44 ± 75.35	249.10 ± 113.38	282.58 ± 170.85	0.788	236.03 ± 86.27	294.75 ± 177.26	252.20 ± 69.48	0.241
Total fiber (g)	23.07 ± 4.91	20.05 ± 5.13	19.40 ± 5.37	0.064	**18.23 ± 4.54 ^a^**	**19.72 ± 5.63 ^ab^**	**23.45 ± 4.49 ^b^**	**0.021**	21.11 ± 4.50	19.82 ± 5.49	19.64 ± 6.64	0.662	19.71 ± 6.33	18.48 ± 4.27	22.89 ± 4.65	0.092
Soluble fiber (g)	7.04 ± 1.81	6.59 ± 1.47	6.89 ± 2.54	0.755	**5.87 ± 1.65 ^a^**	**6.12 ± 1.41 ^a^**	**8.15 ± 2.05 ^b^**	**0.003**	6.68 ± 1.47	6.46 ± 1.66	7.02 ± 2.82	0.820	6.34 ± 2.52	6.21 ± 1.48	7.73 ± 1.23	0.120
Insoluble fiber (g)	**15.86 ± 3.58 ^a^**	**13.17 ± 3.52 ^ab^**	**12.31 ± 3.25 ^b^**	**0.010**	12.20 ± 3.12	13.24 ± 4.41	15.16 ± 3.04	0.075	14.03 ± 3.37	12.89 ± 3.89	12.46 ± 3.96	0.423	12.89 ± 4.12	11.99 ± 2.85	14.87 ± 3.64	0.118
Fructose	**16.09 ± 5.79 ^a^**	**11.06 ± 5.11 ^ab^**	**8.98 ± 4.90 ^b^**	**0.005**	11.87 ± 4.09	11.01 ± 6.61	12.59 ± 6.78	0.598	**12.82 ± 5.37 ^ab^**	**14.92 ± 4.84 ^a^**	**10.75 ± 5.57 ^b^**	**0.043**	12.11 ± 3.90	12.38 ± 6.65	14.91 ± 5.47	0.341
Sucrose	17.44 ± 6.74	13.52 ± 3.76	15.00 ± 5.88	0.116	12.76 ± 5.93	14.48 ± 5.12	17.98 ± 5.39	0.091	15.92 ± 5.21	19.37 ± 12.97	17.15 ± 9.39	0.277	15.18 ± 8.24	17.67 ± 6.38	20.59 ± 13.81	0.147
Vitamin B1 (mg)	0.84 ± 0.15	0.79 ± 0.15	0.73 ± 0.17	0.059	0.71 ± 0.16	0.76 ± 0.17	0.85 ± 0.14	0.082	0.77 ± 0.18	0.72 ± 0.21	0.71 ± 0.17	0.478	0.72 ± 0.16	0.68 ± 0.22	0.82 ± 0.17	0.142
Vitamin B2 (mg)	1.21 ± 0.14	1.31 ± 0.40	1.19 ± 0.29	0.365	1.19 ± 0.26	1.11 ± 0.27	1.37 ± 0.28	0.065	1.16 ± 0.23	1.25 ± 0.32	1.21 ± 0.26	0.790	1.17 ± 0.26	1.15 ± 0.31	1.33 ± 0.21	0.202
Vitamin B6 (mg)	**1.26 ± 0.19 ^a^**	**1.12 ± 0.35 ^ab^**	**0.98 ± 0.29 ^b^**	**0.026**	1.12 ± 0.24	1.04 ± 0.38	1.17 ± 0.27	0.502	1.12 ± 0.23	1.11 ± 0.37	1.06 ± 0.37	0.654	1.13 ± 0.26	0.94 ± 0.33	1.24 ± 0.32	0.057
Folate (µg)	308.67 ± 60.59	306.63 ± 89.42	271.15 ± 88.13	0.148	287.82 ± 59.86	266.37 ± 86.19	319.26 ± 85.06	0.295	283.29 ± 52.20	297.09 ± 84.35	277.33 ± 60.63	0.802	286.49 ± 60.29	263.26 ± 65.39	313.62 ± 71.91	0.084
Vitamin C (mg)	**113.51 ± 36.26 ^a^**	**98.30 ± 40.49 ^ab^**	**74.81 ± 40.41 ^b^**	**0.018**	98.00 ± 21.64	85.66 ± 53.57	97.96 ± 42.70	0.702	102.00 ± 48.91	91.20 ± 32.31	76.44 ± 37.71	0.220	**107.41 ± 42.59 ^a^**	**69.99 ± 37.80 ^b^**	**91.79 ± 31.95 ^ab^**	**0.023**
Sodium (mg)	3667.22 ± 828.11	3964.68 ± 1163.61	4525.68 ± 1110.80	0.197	3589.81 ± 726.56	3869.49 ± 1099.46	4623.72 ± 1101.74	0.080	3570.49 ± 888.08	3972.52 ± 816.14	4082.09 ± 764.50	0.333	3555.95 ± 780.07	3874.52 ± 825.84	4271.26 ± 812.05	0.097
Potassium (mg)	2376.74 ± 401.73	2204.30 ± 587.84	2002.19 ± 501.86	0.083	**2048.94 ± 336.88 ^a^**	**1990.87 ± 501.59 ^b^**	**2429.63 ± 543.72 ^a^**	**0.032**	2161.36 ± 330.12	2219.47 ± 546.81	1957.92 ± 531.25	0.271	2114.59 ± 369.77	1947.93 ± 558.93	2340.03 ± 461.73	0.076
Calcium (mg)	743.34 ± 134.22	834.95 ± 240.63	759.01 ± 184.95	0.423	**749.04 ± 204.82 ^ab^**	**681.99 ± 152.06 ^a^**	**870.18 ± 164.83 ^b^**	**0.023**	712.00 ± 178.07	810.17 ± 196.26	786.39 ± 191.73	0.453	741.32 ± 198.95	718.71 ± 195.02	865.16 ± 142.10	0.145
Magnesium (mg)	268.77 ± 57.04	248.11 ± 58.97	233.62 ± 58.99	0.168	**223.16 ± 54.72 ^a^**	**235.29 ± 54.19 ^ab^**	**278.81 ± 55.27 ^b^**	**0.015**	270.32 ± 60.82	237.91 ± 72.33	232.70 ± 62.61	0.206	246.94 ± 59.53	228.12 ± 79.89	271.00 ± 55.04	0.225
Phosphorus (mg)	1048.64 ± 166.85	1108.31 ± 308.17	1047.14 ± 219.07	0.666	**980.16 ± 212.85 ^a^**	**954.79 ± 171.97 ^a^**	**1214.88 ± 206.92 ^b^**	**0.003**	1038.74 ± 214.05	1032.56 ± 271.81	1043.87 ± 213.43	0.855	991.12 ± 211.91	993.29 ± 283.47	1152.48 ± 154.78	0.234
Iron (mg)	9.07 ± 1.86	8.23 ± 2.15	8.29 ± 1.95	0.294	**7.34 ± 1.67 ^a^**	**8.18 ± 1.75 ^ab^**	**9.66 ± 1.80 ^b^**	**0.005**	8.92 ± 2.67	7.92 ± 2.42	8.12 ± 2.10	0.427	8.11 ± 2.62	8.01 ± 2.71	8.97 ± 1.71	0.537
Zinc (mg)	8.46 ± 1.50	8.93 ± 2.53	8.83 ± 2.33	0.852	**8.05 ± 1.81 ^ab^**	**7.82 ± 1.60 ^a^**	**9.98 ± 2.17 ^b^**	**0.009**	8.31 ± 1.57	8.05 ± 2.10	8.37 ± 2.20	0.677	7.71 ± 1.58	7.80 ± 2.23	9.43 ± 1.53	0.084

Data are presented as mean ± SD. Dietary intake variables were compared across tertiles using one-way ANCOVA adjusted for age and sex. When the overall *p*-value was significant (*p* < 0.05), post hoc pairwise comparisons were conducted using Bonferroni-corrected pairwise ANCOVA. Bold values indicate statistically significant results (*p* < 0.05). Different superscript letters (a, b) indicate statistically significant differences between groups, whereas groups sharing at least one letter are not significantly different.

**Table 7:** The odds of sarcopenia risk factors across tertiles of DII, DIL, DGI, and DGL in diabetic hemodialysis patients (DM+).

	Tertiles	DII Model 1	DII Model 2	DII Model 3	DIL Model 1	DIL Model 2	DIL Model 3	DGI Model 1	DGI Model 2	DGI Model 3	DGL Model 1	DGL Model 2	DGL Model 3
Sarcopenia diagnosis	T1 (Ref)	1	1	1	1	1	1	1	1	1	1	1	1
	T2	1.57 (0.30–8.15)	1.00 (0.15–6.69)	1.23 (0.13–11.39)	1.59 (0.28–8.95)	1.90 (0.28–12.91)	9.30 (0.53–162.72)	1.71 (0.34–8.63)	1.44 (0.24–8.78)	0.93 (0.08–10.84)	2.08 (0.40–10.88)	1.60 (0.25–10.39)	1.41 (0.14–13.86)
	T3	2.05 (0.40–10.42)	1.23 (0.19–8.14)	0.99 (0.08–12.58)	1.96 (0.41–9.42)	2.17 (0.33–14.01)	3.98 (0.38–41.36)	1.03 (0.22–4.79)	0.69 (0.12–4.13)	0.47 (0.03–8.01)	1.19 (0.23–6.16)	0.88 (0.13–5.95)	1.76 (0.18–17.16)
	*p* trend	0.377	0.795	0.988	0.395	0.421	0.235	0.999	0.650	0.577	0.897	0.814	0.625
SGA-7p	T1 (Ref)	1	1	1	1	1	1	1	1	1	1	1	1
	T2	0.57 (0.13–2.57)	0.84 (0.15–4.68)	0.79 (0.13–4.97)	1.57 (0.32–7.65)	1.81 (0.32–10.18)	1.31 (0.20–8.66)	1.78 (0.38–8.47)	2.70 (0.46–15.96)	2.53 (0.37–17.41)	0.23 (0.04–1.18)	0.23 (0.04–1.46)	0.20 (0.03–1.35)
	T3	0.57 (0.13–2.57)	0.92 (0.16–5.21)	0.51 (0.07–3.56)	1.15 (0.28–4.82)	1.33 (0.26–6.93)	0.78 (0.13–4.73)	1.46 (0.35–6.06)	2.42 (0.43–13.50)	1.70 (0.25–11.67)	0.74 (0.16–3.43)	0.90 (0.15–5.31)	0.41 (0.05–3.38)
	*p* trend	0.471	0.948	0.481	0.879	0.743	0.794	0.617	0.332	0.602	0.844	0.916	0.397
SARCF	T1 (Ref)	1	1	1	1	1	1	1	1	1	1	1	1
	T2	0.85 (0.16–4.60)	0.65 (0.05–8.52)	1.71 (0.10–30.60)	0.41 (0.06–2.62)	0.06 (0.00–1.54)	0.03 (0.00–1.84)	2.43 (0.40–14.72)	5.51 (0.40–76.47)	6.12 (0.33–112.72)	0.30 (0.04–2.50)	0.10 (0.00–2.08)	0.24 (0.01–5.98)
	T3	0.85 (0.16–4.60)	0.75 (0.06–10.34)	1.22 (0.06–24.95)	0.53 (0.09–3.06)	0.48 (0.03–7.39)	0.25 (0.01–5.67)	1.74 (0.37–8.14)	7.64 (0.70–83.76)	13.17 (0.42–416.65)	0.23 (0.03–1.75)	0.13 (0.01–2.68)	0.81 (0.02–33.30)
	*p* trend	0.848	0.878	0.942	0.515	0.919	0.941	0.489	0.092	0.125	0.146	0.267	0.591

DII: dietary insulin index, DIL: dietary insulin load, DGI: dietary glycemic index, DGL: dietary glycemic load Model 1: Crude (unadjusted). Model 2: Adjusted for age and sex. Model 3: Adjusted for age, sex, BMI, dialysis vintage, DM duration, CRP. T1 (lowest tertile) was used as the reference category for all indices. The *p* for trend was calculated by entering the tertile variable as a continuous term in the logistic regression model.

**Table 8:** The odds of sarcopenia risk factors across tertiles of DII, DIL, DGI, and DGL in diabetic hemodialysis patients (DM−).

	Tertiles	DII Model 1	DII Model 2	DII Model 3	DIL Model 1	DIL Model 2	DIL Model 3	DGI Model 1	DGI Model 2	DGI Model 3	DGL Model 1	DGL Model 2	DGL Model 3
Sarcopenia diagnosis	T1 (Ref)	1	1	1	1	1	1	1	1	1	1	1	1
	T2	2.17 (0.51–9.12)	2.63 (0.42–16.42)	8.35 (0.28–249.42)	1.60 (0.40–6.42)	0.92 (0.17–4.89)	1.05 (0.11–10.34)	3.71 (0.88–15.61)	2.39 (0.43–13.35)	1.12 (0.09–14.60)	0.60 (0.14–2.55)	0.48 (0.08–2.72)	0.50 (0.04–5.71)
	T3	1.52 (0.36–6.37)	1.98 (0.34–11.35)	1.95 (0.16–23.10)	2.13 (0.48–9.46)	1.34 (0.21–8.62)	0.56 (0.04–7.14)	1.29 (0.26–6.27)	0.44 (0.06–3.04)	0.22 (0.01–3.48)	1.67 (0.41–6.76)	0.98 (0.18–5.22)	1.31 (0.13–13.53)
	*p* trend	0.556	0.478	0.601	0.313	0.778	0.696	0.694	0.456	0.313	0.522	0.909	0.932
SGA-7p	T1 (Ref)	1	1	1	1	1	1	1	1	1	1	1	1
	T2	2.81 (0.68–11.60)	2.10 (0.48–9.30)	1.27 (0.25–6.49)	1.24 (0.33–4.67)	1.52 (0.37–6.24)	1.52 (0.35–6.61)	1.13 (0.30–4.32)	1.25 (0.30–5.22)	1.44 (0.32–6.62)	1.67 (0.43–6.43)	2.12 (0.50–8.92)	1.59 (0.35–7.21)
	T3	2.44 (0.61–9.74)	1.97 (0.47–8.30)	1.83 (0.41–8.29)	3.24 (0.72–14.50)	2.64 (0.54–12.96)	2.68 (0.51–14.02)	2.42 (0.57–10.34)	2.14 (0.46–10.01)	2.17 (0.44–10.61)	2.88 (0.69–12.03)	2.71 (0.58–12.56)	2.35 (0.49–11.22)
	*p* trend	0.197	0.351	0.424	0.131	0.226	0.237	0.239	0.329	0.334	0.142	0.180	0.276
SARCF	T1 (Ref)	1	1	1	1	1	1	1	1	1	1	1	1
	T2	1.01 (0.24–4.27)	1.86 (0.36–9.72)	2.47 (0.31–19.46)	1.00 (0.25–3.96)	0.67 (0.14–3.12)	0.53 (0.10–2.81)	0.72 (0.17–2.98)	0.56 (0.11–2.96)	0.55 (0.09–3.18)	1.29 (0.32–5.17)	1.05 (0.23–4.77)	0.93 (0.18–4.86)
	T3	0.50 (0.11–2.36)	0.64 (0.12–3.53)	1.00 (0.16–6.17)	0.42 (0.07–2.34)	0.54 (0.08–3.69)	0.51 (0.07–3.68)	0.54 (0.11–2.63)	0.68 (0.11–4.14)	0.43 (0.06–3.16)	0.42 (0.08–2.28)	0.40 (0.06–2.67)	0.46 (0.07–3.11)
	*p* trend	0.387	0.669	0.975	0.338	0.484	0.436	0.425	0.645	0.371	0.353	0.384	0.440

DII: dietary insulin index, DIL: dietary insulin load, DGI: dietary glycemic index, DGL: dietary glycemic load. Model 1: Crude (unadjusted). Model 2: Adjusted for age and sex. Model 3: Adjusted for age, sex, BMI, dialysis vintage, CRP. T1 (lowest tertile) was used as the reference category for all indices. The *p* for trend was calculated by entering the quartile variable as a continuous term in the logistic regression model.

## Data Availability

The data presented in this study are available from the corresponding author upon request.

## Abbreviations

The following abbreviations are used in this manuscript:

BIA	Bioelectrical impedance analysis
BMI	Body mass index
BUN	Blood urea nitrogen
CKD	Chronic kidney disease
CRP	C-reactive protein
DGI	Dietary glycemic index
DGL	Dietary glycemic load
DII	Dietary insulin index
DIL	Dietary insulin load
DM	Diabetes Mellitus
ESRD	End-stage renal disease
EWGSOP2	European Working Group on Sarcopenia in Older People criteria
GFR	Glomerular filtration rate
HD	Hemodialysis
HDL	High-density lipoprotein
LDL	Low-density lipoprotein
MHD	Maintenance hemodialysis
MUAC	Mid-upper arm circumference
NIH	National Institutes of Health
PEW	Protein-energy wasting
SGA-7p	Seven-point subjective global assessment
TSF	Triceps skinfold thickness
TIBC	Total iron binding capacity

## Appendix A

**Table A1:** Overall demographic, clinical, anthropometric, and sarcopenia-related characteristics of diabetic and non-diabetic hemodialysis patients.

Variables	DM+ (*n* = 43)	DM− (*n* = 49)	*p*-Value *	*p*-Value ^¥^
Age (year)	60.19 ± 13.03	51.65 ± 18.22	**0.012**	0.602
Gender				
Male (%)	22 (51.2%)	23 (46.9%)	0.845	1.000
Female (%)	21 (48.8%)	26 (53.1%)	0.845	1.000
Marital status				
Single	2 (4.7%)	11 (22.4%)	**0.017**	0.118
Married	41 (95.3%)	38 (77.6%)	**0.017**	0.118
Education Level				
Illiterate	6 (14.0%)	11 (22.4%)	0.863	0.090
Literate (no formal education)	6 (14.0%)	5 (10.2%)	0.863	0.090
Primary school	18 (41.9%)	17 (34.7%)	0.863	0.090
Secondary school	6 (14.0%)	6 (12.2%)	0.863	0.090
High school	5 (11.6%)	8 (16.3%)	0.863	0.090
University	2 (4.7%)	2 (4.1%)	0.863	0.090
Dialysis parameter				
Dialysis duration (years)	6.26 ± 4.82	6.02 ± 5.08	0.810	0.916
Anthropometric measurements				
Weight (kg)	71.33 ± 14.81	67.59 ± 14.10	0.218	0.445
MUAC (cm)	26.93 ± 5.17	25.94 ± 4.57	0.404	0.649
TSF (mm)	17.50 ± 7.79	15.86 ± 6.63	0.610	0.681
Waist circumference (cm)	90.19 ± 15.00	88.48 ± 12.25	0.550	0.938
Hip circumference (cm)	91.45 ± 14.70	90.39 ± 11.48	0.953	0.742
Waist/hip ratio	0.99 ± 0.05	0.98 ± 0.06	0.503	0.912
Body fat (%)	28.71 ± 8.06	26.69 ± 10.02	0.292	0.361
Lean mass (kg)	22.34 ± 3.95	21.34 ± 3.46	0.198	0.254
BMI (kg/m^2^)	26.10 ± 5.35	25.04 ± 5.10	0.336	0.577
Sarcopenia related measurements				
Hand grip strength (kg)	**17.99 ± 4.16**	**20.22 ± 4.75**	**0.020**	**0.011**
Walking time (s)	**8.00 ± 2.60**	**5.92 ± 1.51**	**<0.001**	**<0.001**
Walking speed (m/s)	**0.56 ± 0.20**	**0.72 ± 0.20**	**<0.001**	**<0.001**
SARC-F total score	**5.33 ± 2.74**	**2.82 ± 2.14**	**<0.001**	**<0.001**
Total SQL score	44.16 ± 6.01	48.60 ± 9.19	0.184	0.217
SGA-7p score	6.09 ± 1.09	6.24 ± 0.90	0.538	0.950
SGA-7p category				
Well-nourished	34 (79.1%)	40 (81.6%)	0.560	0.958
Mild to moderate malnutrition	8 (18.6%)	9 (18.4%)	0.560	0.958
Severe malnutrition	1 (2.3%)	0 (0.0%)	0.560	0.958
Sarcopenia diagnosis				
Present, n (%)	14 (32.6%)	18 (36.7%)	0.841	0.138
Absent, n (%)	29 (67.4%)	31 (63.3%)	0.841	0.138

Data are presented as mean ± SD for continuous variables and *n* (%) for categorical variables. *p*-value * Unadjusted comparison between DM+ and DM− groups (*t*-test or Mann–Whitney U for continuous variables; chi-square or Fisher’s exact test for categorical variables). Normality was assessed using the Shapiro–Wilk test. *p*-value ^¥^ Age and gender-adjusted comparison between DM+ and DM− groups (ANCOVA or rank-based ANCOVA for continuous variables; logistic regression for categorical variables). Bold values indicate statistically significant results (*p* < 0.05).

**Table A2:** Overall biochemical parameters of diabetic and non-diabetic hemodialysis patients.

Variables	DM+ (*n* = 43)	DM− (*n* = 49)	*p*-Value *	*p*-Value ^¥^
Albumin (g/dL)	38.93 ± 2.93	40.46 ± 2.87	**0.014**	0.097
Total protein (g/dL)	66.42 ± 7.35	68.17 ± 13.82	0.988	0.931
Fasting glucose (mg/dL)	**212.72 ± 71.27**	**113.36 ± 25.78**	**<0.001**	**<0.001**
BUN before (mg/dL)	137.98 ± 27.70	138.80 ± 26.04	0.885	0.802
BUN after (mg/dL)	37.60 ± 13.49	37.31 ± 12.98	0.916	0.985
Creatinine before (mg/dL)	7.87 ± 1.31	8.17 ± 1.60	0.320	0.344
Creatinine after (mg/dL)	2.70 ± 0.60	2.80 ± 0.70	0.469	0.394
Uric acid (mg/dL)	5.92 ± 0.84	6.10 ± 0.78	0.126	0.071
Calcium (mg/dL)	8.73 ± 0.40	8.63 ± 0.51	0.293	0.173
Phosphorus (mg/dL)	4.77 ± 1.11	4.97 ± 1.11	0.420	0.950
Sodium (mEq/L)	137.94 ± 2.26	137.20 ± 2.15	**0.036**	0.223
Potassium before (mEq/L)	5.44 ± 0.77	5.38 ± 0.74	0.500	0.657
Potassium after (mEq/L)	3.69 ± 0.30	3.59 ± 0.28	0.077	0.158
CRP (mg/L)	9.69 ± 10.41	10.51 ± 13.09	0.836	0.738
Ferritin (ng/mL)	627.86 ± 277.31	608.55 ± 223.00	0.851	0.852
TIBC (µg/dL)	156.53 ± 58.10	159.48 ± 59.74	0.910	0.608
HDL (mg/dL)	39.70 ± 11.81	39.41 ± 14.06	0.479	0.625
LDL (mg/dL)	90.86 ± 22.19	84.59 ± 25.11	0.062	0.061
Triglycerides (mg/dL)	141.49 ± 78.80	135.21 ± 73.27	0.842	0.512
Total cholesterol (mg/dL)	160.13 ± 30.87	152.32 ± 28.76	0.213	0.285
Kt/V	1.60 ± 0.28	1.63 ± 0.38	0.793	0.422

Data are presented as mean ± SD for continuous variables and *n* (%) for categorical variables. *p*-value * Unadjusted comparison between DM+ and DM− groups (independent samples *t*-test for normally distributed variables; Mann–Whitney U test for non-normally distributed variables; chi-square or Fisher’s exact test for categorical variables). Normality was assessed using the Shapiro–Wilk test. *p*-value ^¥^ Adjusted comparison between DM+ and DM− groups, controlling for age and gender (ANCOVA for continuous variables with normally distributed residuals; rank-based ANCOVA for non-normally distributed residuals; logistic regression for categorical variables). Bold values indicate statistically significant results (*p* < 0.05).

**Table A3:** Overall dietary energy and nutrient intake of diabetic and non-diabetic hemodialysis patients.

Variables	DM+ (*n* = 43)	DM− (*n* = 49)	*p*-Value *	*p*-Value ^¥^
Energy (kcal)	1672.44 ± 272.45	1679.68 ± 297.26	0.975	0.906
Carbohydrate (g)	204.28 ± 41.84	203.23 ± 44.34	0.908	0.623
Protein (g)	62.65 ± 14.47	61.20 ± 14.60	0.634	0.544
Protein (g/kg)	0.90 ± 0.23	0.95 ± 0.29	0.458	0.863
Total fat (g)	65.21 ± 12.34	67.15 ± 15.23	0.508	0.507
PUFA (g)	12.49 ± 4.33	13.22 ± 5.69	0.625	0.657
MUFA (g)	24.23 ± 5.42	25.58 ± 7.06	0.312	0.287
Saturated fat (g)	23.88 ± 5.14	23.45 ± 5.37	0.702	0.681
Cholesterol (mg)	238.00 ± 126.85	259.82 ± 120.69	0.277	0.375
Total fiber (g)	20.78 ± 5.29	20.22 ± 5.46	0.620	0.415
Soluble fiber (g)	6.86 ± 2.02	6.70 ± 1.98	0.693	0.602
Insoluble fiber (g)	13.71 ± 3.70	13.16 ± 3.72	0.482	0.313
Fructose	11.88 ± 5.99	13.00 ± 5.40	0.347	0.734
Sucrose	15.38 ± 5.78	17.54 ± 9.73	0.278	0.515
Vitamin B1 (mg)	0.78 ± 0.16	0.73 ± 0.19	0.196	0.165
Vitamin B2 (mg)	1.23 ± 0.29	1.21 ± 0.27	0.676	0.608
Vitamin B6 (mg)	1.11 ± 0.30	1.10 ± 0.32	0.834	0.742
Folate (µg)	293.27 ± 80.73	286.65 ± 67.04	0.669	0.964
Vitamin C (mg)	93.96 ± 41.71	90.73 ± 40.71	0.708	0.666
Sodium (mg)	4089.62 ± 1085.27	3864.35 ± 840.17	0.266	0.218
Potassium (mg)	2180.54 ± 512.12	2124.58 ± 480.23	0.590	0.566
Calcium (mg)	775.10 ± 187.72	769.32 ± 189.84	0.884	0.942
Magnesium (mg)	249.11 ± 58.90	247.67 ± 66.56	0.913	0.910
Phosphorus (mg)	1064.70 ± 229.19	1037.93 ± 231.94	0.580	0.587
Iron (mg)	8.53 ± 1.97	8.32 ± 2.42	0.661	0.662
Zinc (mg)	8.74 ± 2.12	8.23 ± 1.93	0.231	0.164

Data are presented as mean ± SD for continuous variables and *n* (%) for categorical variables. *p*-value * Unadjusted comparison between DM+ and DM− groups (independent samples *t*-test for normally distributed variables; Mann–Whitney U test for non-normally distributed variables; chi-square or Fisher’s exact test for categorical variables). Normality was assessed using the Shapiro–Wilk test. *p*-value ^¥^ Adjusted comparison between DM+ and DM− groups, controlling for age and gender (ANCOVA for continuous variables with normally distributed residuals; rank-based ANCOVA for non-normally distributed residuals; logistic regression for categorical variables).
